# T-2 Toxin Neurotoxicity: Molecular Mechanisms and Emerging Chemoprotective Strategies

**DOI:** 10.3390/antiox15030278

**Published:** 2026-02-24

**Authors:** Chunyan Xu, Gea Oliveri Conti, Shusheng Tang, Jianzhong Shen, Chongshan Dai

**Affiliations:** 1 College of Veterinary Medicine, Henan Agricultural University, Zhengzhou 450046, China; 2 Department of Medical Sciences, Surgical and Advanced Technologies “G.F. Ingrassia”, Environmental and Public Health Section, University of Catania, I-95123 Catania, Italy; 3 State Key Laboratory of Veterinary Public Health and Safety, College of Veterinary Medicine, China Agricultural University, Beijing 100193, China

**Keywords:** mycotoxins, T-2 toxin, neurotoxicity, molecular mechanisms, neuroprotection

## Abstract

Mycotoxins can contaminate food and raw food materials and are a threat to animal and human health. T-2 toxin is the most toxic secondary metabolite mainly produced by Fusarium species among trichothecenes. T-2 toxin exposure can induce multiple toxic effects, including hepatotoxicity, nephrotoxicity, immunotoxicity, gastrointestinal toxicity, and reproductive toxicity. Recent studies have reported that T-2 toxin can cross the blood–brain barrier and trigger neurotoxicity. In this review, we summarized the neurotoxic effects caused by T-2 toxin exposure and the underlying molecular mechanisms. Additionally, effective neuroprotective agents, potential clinical applications, and future prospects are discussed. The current studies revealed that the molecular mechanisms of T-2 toxin-induced neurotoxicity involve oxidative stress, mitochondrial dysfunction, neuroinflammation, autophagy, ferroptosis and cell apoptosis. Several signaling pathways, including NFE2L2, NRF-2, PGC-1, p53, BTG2, AKNA, MAPK, Akt, mTOR, HMGB1, HIF-1, CREB, and NF-κB, are involved. Additionally, it was reported that several antioxidants, small inhibitors and nature products, such as daucosterol, betulinic acid, AHN 1-055 hydrochloride, dimethyl fumarate and minocycline supplementations, can partly ameliorate these harmful effects. This review provides valuable insights into the underlying mechanisms of T-2 toxin-induced neurotoxicity and novel effective detoxification strategies.

## 1. Introduction

Mycotoxins are structurally diverse secondary metabolites produced by filamentous fungi, such as Fusarium, Penicillium, and Aspergillus. Worryingly, mycotoxins commonly contaminate the human food chain across various grains, food, fruits, nuts, Chinese herbal medicines, drinking water, and feed, posing a threat to animal and human health [1,2,3,4,5,6]. Currently, due to climate change and the development of detection technology, the contamination rate of mycotoxins has increased to 60–80%, far exceeding 25% in the 1980s. This figure may be even higher in low-income countries [7,8,9]. In addition, the diversity of mycotoxins and the multifaceted toxic effects further exacerbate the difficulty of preventing and controlling fungal toxins.

To date, more than about 700 mycotoxins have been detected and identified in food and food raw materials [10,11]. Of these identified mycotoxins, many mycotoxins exhibit potent toxic effects to humans and animals even at low levels, and therefore pose a serious threat to public health and food security [5,12]. For example, aflatoxin B1 (AFB1), ochratoxin A (OTA), and fumonisin B1 (FB1) were classified as human Groups 1, 2B, and 2B carcinogens, respectively, by the WHO International Agency for Research on Cancer [13]. Epidemiological studies have shown a positive correlation between exposure to certain mycotoxins and the occurrence of infertility, cardiovascular disease and cancer [14,15,16,17].

**Figure 1:**
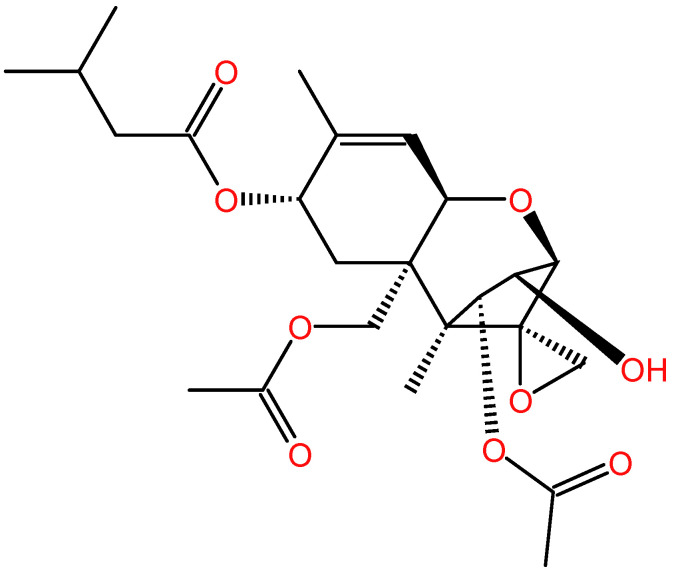
T-2 toxin’s chemical structure.

T-2 toxin (Figure 1) is the most toxic secondary metabolite among trichothecenes, mainly produced by Fusarium species, which commonly occur typically in various food crops, such as barley, wheat, maize, rice, oat, and animal feed in the field or during storage [18]. Recent studies showed that T-2 toxin is also detected in drinking water in endemic areas of China (e.g., Qinghai and Sichuan Provinces) and in traditional Chinese medicines [19,20,21,22]. A recent epidemiological investigation showed that the contents of T-2 toxin in brick tea are positively related with the occurrence and development of Kashin–Beck disease in Tibet, China [23]. It poses a high risk in the field of public health and safety.

Over the past forty years, the toxicology files of T-2 toxin have been extensively studied. T-2 toxin exhibits a potent acute toxicity, and the lethal doses of 50% of T-2 toxin via the intravenous injection at a signal dose in pigs and rats are 1.21 and 0.9 mg/kg body weight, respectively [24,25,26]. The exposure of T-2 toxin and its metabolic derivatives (such as T-2 toxin and T-2-glucoside A) to rodents can induce hepatoxicity, nephrotoxicity, immunotoxicity, reproductive toxicity, cardiac toxicity, skin toxicity and gastrointestinal toxicity [27,28,29,30,31,32,33].

T-2 toxin can also cross the blood–brain barrier and enter brain tissues, then induce brain injury, finally culminating in neurotoxicity [34,35,36]. Animal studies found that T-2 toxin exposure can induce anorexia and decrease learning and memory [37,38]. Mechanistic investigations revealed that T-2 toxin-mediated neurotoxicity involves mitochondrial dysfunction, oxidative stress, neuroinflammation, autophagy, pyroptosis, ferroptosis and cell apoptosis [37,38,39]. Several signaling pathways, including NFE2L2, NRF-2, PGC-1, p53, BTG2, AKNA, MAPK, Akt, mTOR, HMGB1, CREB, and NF-κB, are known to be involved. In the present review, we utilized keywords such as “T-2 toxin” and “neurotoxicity” or “neurotoxic effects” to gather research on the neurotoxic effects of T-2 toxin and the underlying molecular mechanisms from databases such as Web of Science and PubMed, from 1980 up to December 2025. The collected information was then summarized and discussed. Additionally, we discuss effective neuroprotective agents and their clinical application. We hope this review can provide valuable insights into the underlying causes of T-2 toxin-induced neurotoxicity and effective detoxification strategies.

## 2. T-2 Toxin’s Metabolism and the Accumulation in Brain Tissues

Typically, ingestion serves as the main route of T-2 toxin poisoning. Once ingested, it is absorbed across the gastrointestinal tract and enters the bloodstream. In mammals, the biotransformation of T-2 toxin mainly occurs in liver tissue, and this process involves conjugation, deoxidation, and hydroxylation reactions [40]. Correspondingly, it is metabolized to T-2 triol, 3′-OH-HT-2, 3′-OH-T-2, HT-2, and neosolaniol (NEO) [41,42,43,44,45]. Early toxicological studies reported that the half-lives of T-2 and HT-2 toxins are approximately 21 and 73 min after intramuscular injection in dogs. In whole blood, HT-2 and T-2 toxins were both unstable and can be degraded by carboxyl-esterase enzymes in the red blood cells, and the corresponding stability half-lives were 0.84 and 6.9 h, respectively [46,47,48]. In human primary astrocytes, T-2 toxin can be quickly uptaken then subjected to metabolism, leading to HT-2 toxin [36].

Guo et al. found that, when rats were orally given T-2 toxin at 2 mg/kg body weight, low concentrations of T-2 toxin could be detected in the brain tissues on the 1st day, but it was undetectable on the 3rd and 7th days [49]. This may be relative to the stability of T-2 toxin. The C-3′ hydroxylation of T-2 and HT-2 toxins can be mainly catalyzed by intracellular cytochrome P450 enzymes (CYP450s), such as CYP1A1, CYP1A2, CYP2J2, CYP2E1, CYP3A4, CYP3A9, CYP3A11 and CYP3A13. In general, these CYP450s are mainly expressed in the liver tissues. Recent studies showed that they are also expressed in the brain tissues of both rodents and humans, although the levels are low [50,51,52,53,54]. This indicated that T-2 toxin can be catalyzed in brain tissues. Consistently, it was reported that a higher dose of T-2 toxin (i.e., at 100 ng/mL) can significantly upregulate the expression of CYP4501A1 and CYP4503A via the activation of aromatic hydrocarbon receptors (AhR) and then can conversely regulate the metabolism of T-2 toxin [55]. This information indicated that T-2 toxin can be metabolized in brain tissues. This can also explain the detected low level of T-2 toxin in the brain tissues. However, the precise mechanisms are still unclear and require further exploration.

Additionally, with the development of technology, the detection of T-2 toxin is becoming increasingly sensitive, and the minimum detection limit can reach 0.1 ng/mL in human urine samples [56,57]. A recent study investigated the levels of T-2 toxin, T-2 Toxin-3-Glucoside and their metabolites in human urine samples (a total of 300 samples) in South Italy using a high-resolution mass spectrometry method. It found that almost all the major T-2 metabolites can be detected, including 3′-OH-T-2, T-2 triol, HT-2, NEO, HT-2-3-GlcA, T-2-3-GlcA, and HT-2-4-GlcA. The levels of 3′-OH-T-2, T-2 triol, HT-2, NEO, and HT-2-3-GlcA are high, and they can be potential monitoring biomarkers. T-2 was quantified in 21% of samples at a mean concentration of 1.34 ng/mg creatinine [56].

Currently, toxicokinetic or biomonitoring studies of T-2 toxin and metabolites in humans are severely lacking, which significantly limits effective risk assessment. More research is still needed in the future.

## 3. An Overview of T-2 Toxin Exposure-Induced Neurotoxic Effects

In humans, exposure to T-2 toxin is primarily from the ingestion of contaminated cereals and grains or the food chain [58]. Given the ubiquitous occurrence of Fusarium species across agricultural landscapes, there is a possibility of persistent low-level exposure for the global population. A recent epidemiological investigation suggested a plausible connection between mycotoxin contamination and an elevated susceptibility to neurodegenerative conditions such as Alzheimer’s and Parkinson’s diseases [59]. Nevertheless, these analyses frequently encounter limitations due to confounding elements, including the co-occurrence of multiple mycotoxins and other environmental contaminants, which complicates the causation of T-2 toxin-caused toxic effects in humans.

The neurobehavioral effects of T-2 toxin exposure range from acute neurological deficits to chronic behavioral problems [26,37,38,49,60,61,62,63]. For example, in rodent studies, acute T-2 toxin exposure results in vomiting, rapid heart rate, diarrhea, a lack of coordination, muscle weakness, a loss of appetite, depression, and reduced movement [26,37,38,60,61,62,63,64]. Guo et al. found that, when rats were given T-2 toxin orally at a dose of 2 mg/kg body weight, it caused marked brain damage and abnormal neurological responses (e.g., fear) [49]. Similarly, Li et al. showed that T-2 toxin treatment via an intraperitoneal injection at 4 mg/kg body weight can significantly reduce spatial learning, memory, and movement abilities in a mouse model [37]. More recently, Chen et al. reported that T-2 toxin treatment at 1.5 mg/kg body weight every day for 14 days via oral administration can induce depressive-like behaviors such as feeling hopeless and losing interest in pleasurable activities, without showing anxiety [65]. In addition, an exposure to T-2 toxin caused obvious histopathological changes in the brain tissues of mice [49,64,66]. Maroli and his colleagues found that, when T-2 toxin was injected intravenously at 2, 4, or 6 μg/kg body weight, it led to gliosis with acute inflammatory infiltrates in the cerebral and hippocampal tissues. It also damaged glial cells and caused neuroepithelial cell apoptosis [66]. Similarly, Guo et al. found that, when rats were exposed to T-2 toxin orally at 2 mg/kg body weight, it caused bleeding and damage to the brain tissue [49]. Pei et al. showed that, when rats were administrated orally with T-2 toxin at 1–2 mg/kg every day for 28 days, serious hippocampal damage, with disordered cell arrangements and neuronal degeneration, was observed in the brain tissues [67]. Moreover, T-2 toxin can harm the vascular system (such as microvascular dilation and swelling) in brain tissues [49,68,69,70,71,72,73,74,75,76,77]. Table 1 summarizes the various T-2 toxin exposure-caused neurotoxic effects in vivo and in vitro.

## 4. Roles of Neurotransmitters in T-2 Toxin-Induced Neurotoxicity

An exposure to T-2 toxin can have a significant impact on central nervous system (CNS) neurotransmitter levels. For instance, Chi et al. reported that T-2 toxin significantly increased the levels of dopamine (DA) and norepinephrine in chicken brain tissue [78]. This suggests that changes in catecholamines may predominantly contribute to the neurotoxicity induced by T-2 toxin. On the contrary, Huang et al. found that a single intraperitoneal injection of T-2 toxin at a dose of 4 mg/kg body weight significantly decreased the levels of DA, 5-HT, and acetylcholine (Ach) in mouse brain tissue [74]. More recently, Chen et al. observed that an exposure to T-2 toxin significantly decreased the levels of DA by increasing the expression of the dopamine transporter in the nucleus accumbent of the male mouse brain, which then triggers depressive-like behavior [65]. Additionally, Wang et al. showed that the oral administration of T-2 toxin at doses of 0.1, 1, and 2.5 mg/kg body weight can dose-dependently perturb the levels of 5-HT, 5-hydroxyindole acetic acid (5-HIAA), norepinephrine, and DA in specific rat brain regions within 10 h. Notably, these authors reported a significant increase in 5-HT at 2 h after dosing in the nucleus raphe magnus and locus coeruleus across all treatment groups and an increase in DA at 6 h in the locus coeruleus and hypothalamic paraventricular nucleus [79]. This suggests that the effects of T-2 toxin on neurotransmitters in different brain regions may be diverse and varied.

It is known that cholinergic and glutamatergic receptors in the subgranular zone are important for maintaining the proper proliferation and differentiation of granule cell lineages in the hippocampal formation of the brain [80,81,82]. It was reported that T-2 toxin exposure in the pregnant mice significantly decreased the transcript levels of cholinergic and glutamate receptor subunits (i.e., cholinergic receptor nicotinic beta 2 subunit [chrnb2], cholinergic receptor nicotinic alpha 4 subunit [chrna4], and glutamate receptor 2 [gria2]) and glutamate transporters (i.e., solute carrier family 17 member 6 [Slc17a6]) in the dentate gyrus in the offspring, indicating decreased cholinergic signals on hilar GABAergic (which produce gamma-aminobutyric acid [GABA]) interneurons, innervating type-2 cells and decreasing glutamatergic (which produce glutamate) signals in type-1 and type-2 cells [81]. This information indicated that T-2 toxin exposure in pregnant mice may disturb the production of GABA and glutamate, thus affecting the development of offspring hippocampal tissue via disturbing glutamatergic and GABAergic pathways. However, the precise molecular mechanisms remain unclear, and further investigations are required.

Overall, this collective evidence indicates that the effects of T-2 toxin on neurotransmitters are complex and depend on various factors. Of note, the changes in several neurotransmitters including DA, 5-HT, and Ach are various, and this may be dependent on the dosage, animal species, exposure time, and brain region. It may also affect glutamatergic and GABAergic pathways to disturb the development of offspring hippocampal tissue. To date, these precise molecular mechanisms remain unclear, and further investigations are still required.

## 5. Molecular Mechanisms of T-2 Toxin-Induced Neurotoxicity

The fundamental molecular pathways associated with T-2 toxin-induced neurotoxicity have been comprehensively investigated, and key pathways include the overproduction of reactive oxygen species (ROS), oxidative stress, inflammatory responses, mitochondrial dysfunction, apoptosis and autophagy. The aforementioned cellular events are driven by various signaling mechanisms, such as nuclear factor erythroid 2-related factor 2 (NFE2L2), NRF-2, p53, PGC-1α, HIF-1, p53, MAPK, serine/threonine protein kinase/mammalian target of rapamycin (Akt/mTOR), HMGB1, CREB, and NF-κB. In the following section, we will discuss these elements in depth.

### 5.1. Role of Oxidative Stress

The excessive production of reactive oxygen species (ROS) can induce oxidative stress damage. In vitro and in vivo studies have shown that T-2 toxin can cause oxidative stress damage in nerve cells (including neuronal cells or glial cells) or brain tissues [12,49,67,70,83,84,85]. Zhang et al. found that exposing mouse N2a cells to 5–80 ng/mL of T-2 toxin for 24 h can significantly increase the levels of intracellular ROS and malondialdehyde (MDA), a lipid peroxidation biomarker [12,66]. T-2 toxin can also downregulate the activities of several intracellular antioxidant enzymes, such as SOD and CAT, and the levels of GSH [12]. Consistently, it was reported that exposing PC12 or microglial cells to T-2 toxin can significantly increase intracellular ROS and MDA levels and significantly decrease the activities of SOD and CAT and the levels of GSH [70,72]. Similarly, T-2 toxin treatment at 1.57 mg/kg body weight via subcutaneous administration significantly decreased the activities of SOD and CAT and levels of GPX in the brain tissues of mice at the 1st day after administration, and an opposite trend was found in the experiment at the 7th day [61]. Huang et al. showed that a single intraperitoneal injection of 4 mg/kg body weight of T-2 toxin significantly increased ROS and MDA levels and decreased the activities of CAT and GPX and GSH levels in mouse brain tissues [74]. These data suggest that an imbalance of the intracellular antioxidant system was involved in T-2 toxin exposure-induced neurotoxicity.

Similar to ROS, the production of reactive nitrogen species (RNS) also contributes to oxidative stress in brain tissues. RNS include nitric oxide (NO) and its derivatives, such as peroxynitrite (ONOO−), which can cause DNA damage [86]. Pei et al. found that treating PC12 cells with 3–12 ng/mL of T-2 toxin for 24 h can significantly upregulate the expression of NF-κB and iNOS proteins, ultimately promoting the production of NO [70]. This indicates that the activation of the NF-κB/iNOS/NO signaling pathway also partly contributes to T-2 toxin-induced oxidative stress and neurotoxicity. In addition, different models of oxidative stress have been studied to clarify the effects of oxidative stress on NF-κB-related activities [87].

Supplementation with N-acetylcysteine (NAC), an aminothiol and synthetic precursor of intracellular cysteine and GSH, significantly reduces ROS production, effectively alleviating T-2 toxin-induced lipid peroxidation, oxidative damage and cytotoxicity in N2a neuronal cells [12]. Moreover, a recent study revealed that exposing GH3 cells to 10 or 40 nM of T-2 toxin significantly decreased the expression of peroxiredoxin 4 (PRDX4) protein; furthermore, the overexpression of PRDX4 significantly promoted T-2 toxin-induced ROS production, mitochondrial dysfunction and cell apoptosis [88]. PRDX4 is the only secreted antioxidant enzyme in the peroxidase family capable of converting H_2_O_2_ into harmless O_2_ to mitigate oxidative stress. It also plays a crucial role in essential biological processes, including protein folding, DNA repair, inflammatory regulation and tumor development [89]. These findings suggest that T-2 toxin-induced oxidative stress may be partly due to its inhibition of PRDX4 enzyme expression.

Nuclear factor erythroid 2-related factor 2 (NFE2L2) is a crucial transcription factor in the antioxidant defense system [90,91]. It can transcriptionally regulate the expression of various antioxidant genes, including those encoding SOD, CAT, GPX, and heme oxygenase 1 (HO-1) [90]. Previous studies have demonstrated that NFE2L2 is essential for mitigating oxidative damage caused by various toxic compounds, such as cadmium, aflatoxin B1 and cisplatin [3,92,93]. Consistently, Zhang et al. found that exposing mouse neuronal N2a cells to 5–80 ng/mL of T-2 toxin for 24 h can dose-dependently inhibit the expression of NFE2L2 protein and its downstream protein HO-1 [12]. In mice treated with 5.94 mg/kg of T-2 toxin, the mRNA levels of NFE2L2 and the phase II detoxifying enzymes NQO-1, GCLC, GCLM, and HO-1 were significantly decreased on the 1st, 3rd, and 7th days after percutaneous treatment [61]. In another study, it was found that treating SH-SY5Y cells with 5 or 10 ng/mL of T-2 toxin for 6 h significantly upregulated the expression of NFE2L2 protein [83]. Huang et al. found that treating GH3 cells with T-2 toxin also significantly increased the mRNA expression of the NFE2L2 gene [94]. These findings indicate that the expression of NFE2L2 in response to T-2 toxin-induced oxidative stress damage in neuronal cells is context dependent. Consistently, the pharmacological inhibition or gene knockout of NFE2L2 was shown to exacerbate T-2 toxin-induced neuronal cell death, confirming the survival-promoting role of NFE2L2 in T-2 toxin-induced cytotoxicity in neuronal cells [12,94].

Pei et al. showed that silencing the HMGB1 gene significantly inhibits the production of ROS and MDA, increases the activity of SOD, and then alleviates mitochondrial dysfunction and caspase -9, -3-dependent apoptosis [70]. Furthermore, it was found that silencing the HMGB1 gene significantly reduces the expression of the NFE2L2 protein in T-2 toxin-treated PC12 cells [70]. This indicates that HMGB1 may be a key target for intervening in T-2 toxin-induced neurotoxicity.

**Figure 2:**
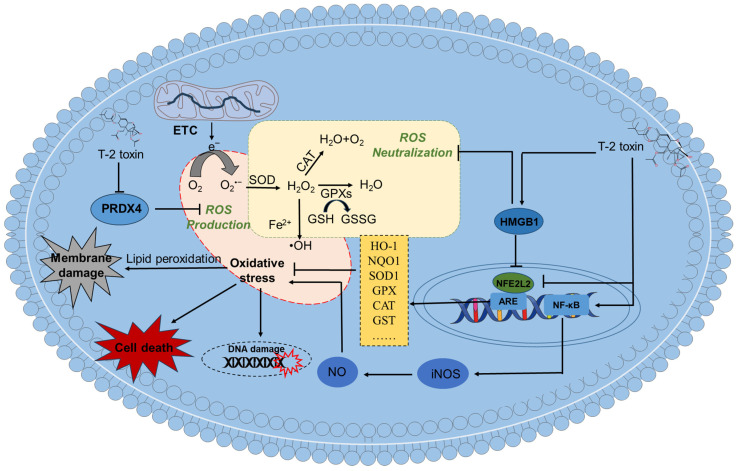
Schematic diagram of the mechanism of action for T-2 toxin-induced oxidative stress in neuronal cells. T-2 toxin exposure can induce lipid peroxidation and the production of ROS production, and can finally induce cell death. It also decreases the activities of SOD, CAT, and GPX, or inhibits the expression of PRDX4, then damages the intracellular antioxidant system. T-2 toxin also induces the production of NO via the activation of NF-κB/iNOS pathway. It can also inhibit the transcriptional expression of NFE2L2, then reduce the expression of HO-1, NQO1, SOD1, GPX, CAT, and GST, finally exacerbating oxidative damage.

In summary, these studies indicate that T-2 toxin exposure can cause oxidative stress damage in nerve cells by inducing ROS and RNS production and downregulating the intracellular antioxidant system, including a decrease in antioxidant enzyme activity and antioxidant content. T-2 toxin exposure can also affect the NFE2L2 pathway, thereby regulating the intracellular antioxidant reduction system. The activation of NFE2L2 can protect against T-2 toxin-induced oxidative stress damage and neurotoxicity. As shown in Figure 2, the current evidence suggests that T-2 toxin-induced oxidative stress in neuronal cells is mainly due to lipid peroxidation, disruption of the body’s antioxidant system, the inhibition of peroxidase expression and the dysregulation of the NFE2L2 pathway.

### 5.2. Role of Mitochondrial Dysfunction and Apoptosis in T-2 Toxin Neurotoxicity

Mitochondria are both generators and targets of ROS [95,96]. Excessive ROS production can disrupt mitochondrial function and lead to cell death. Several studies have shown that an exposure to T-2 toxin can cause mitochondrial dysfunction, resulting in neuronal cell apoptosis [12,67,70,71,94]. Noticeable mitochondrial damage, such as mitochondrial swelling, vacuole formation, and a loss of cristae, has been observed in neuronal cells or brain tissues exposed to T-2 toxin [62,97]. Bin-Umer et al. reported that several trichothecenes, including T-2 toxin, can directly hinder mitochondrial translation [98]. Wan et al. observed that 40 nM of T-2 toxin reduces the expression of heat shock proteins Hsp60 and Hsp70 in rat GH3 cells [99]. Hsp60, a mitochondrial-specific chaperone protein, coordinates the import and folding of cytoplasmic proteins within mitochondria [100,101]. Importantly, the inhibition of Hsp70 impairs mitochondrial proteostasis and function [102]. Thus, the suppression of Hsp60/Hsp70 induced by T-2 toxin likely explains its disruptive effects on mitochondrial genomics (transcription/replication) and the translational mechanism. Consistently, it was also reported that an exposure to T-2 toxin can significantly increase the expression of critical transcription factors and co-activators that control mitochondrial biogenesis, mt-DNA transcription, and replication in GH3 cells. These include mitochondrial transcription factor A (mtTFA), mtTFB1, mtTFB2, estrogen-related receptor alpha (Err-α), nuclear respiratory factors 1 and 2 (NRF-1 and NRF-2), peroxisome proliferator-activated receptor gamma co-activator-related protein 1 (PPRC1), peroxisome proliferative-activated receptor gamma co-activator 1 alpha (PGC-1α), and PGC-1β [94]. In another study, it was reported that treatment with 0.5 ng/mL of T-2 toxin for 72 h or 120 h significantly reduces the expression of PPRC1, PGC-1α, nuclear respiratory factor 1 (NRF-1), and mitochondrial transcription factor A (mtTFA) in murine embryonic stem cells [103]. These different changes may depend on the treatment dose and time of T-2 toxin and the cell type.

The mitochondrial oxidative phosphorylation (OXPHOS) system is the metabolic center of eukaryotic cells, driving the synthesis of ATP. It has been shown that an exposure to T-2 toxin can significantly increase the activity of mitochondrial complex I and the expression of most mitochondrial ETC core subunits (including NADH dehydrogenase iron–sulfur protein 1 [Ndufs1], Ndufs3, Ndufs4, Ndufs6, Ndufs7, Ndufs8, NADH dehydrogenase flavoprotein 1 [Ndufv1], Ndufv2, Ndufv3, NADH dehydrogenase alpha subcomplex assembly factor 1 [Ndufaf1], Ndufaf2, Ndufaf3, Ndufaf4, NADH dehydrogenase subunit 6, mitochondrial [ND6], succinate dehydrogenase flavoprotein subunit A [Sdha], Sdhb, cytochrome c-1, heme protein [Cyc1], rieske iron-sulfur polypeptide 1 [Uqcrfs1], cytochrome c oxidase subunit 1, mitochondrial [COX1], COX2, COX3, ATP synthase F0 subunit 6, mitochondrial [mt-Atp6] and mt-Atp8) in GH3 cells [94]. Furthermore, it has been demonstrated that this upregulation may be due to the activation of nuclear respiratory factor 2α (NRF-2α) [104]. Notably, knocking down NRF-2α significantly reduces the expression of Ndufs3, Ndufs7, Ndufaf1, Ndufaf2, Ndufaf4, mtTFA, mtTFB2, and Drp1 mRNAs in T-2 toxin-treated GH3 cells, promotes the production of ROS, eliminates the increase in ATP and mitochondrial complex I activity induced by T-2 toxin and inhibits the mitochondrial DNA copy number [104]. This indicates that the activities of ETCs, mitochondrial biosynthesis, and mitochondrial dynamics mediated by NRF-2α play a protective role.

Mitochondria maintain the dynamic balance of the mitochondrial network through continuous division and fusion, a process known as mitochondrial dynamics. This is an important basis for maintaining mitochondrial morphology, distribution, and quantity and ensuring cellular homeostasis. In addition, the body degrades dysfunctional mitochondria inside the cell through mitochondrial autophagy, maintaining mitochondrial homeostasis [105]. Guo et al. found that exposing GH3 cells to 40 nM of T-2 toxin for 24 h significantly increases the mRNA expression of Drp1, Fis1, Mfn1, and Opa1 genes, which are involved in mitochondrial dynamics [104]. Additionally, treatment with T-2 toxin can activate mitophagy, as evidenced by the increased expression of the mitophagy-specific proteins NIP-like protein X (NIX), PTEN-induced putative kinase protein 1 (PINK1), and E3 ubiquitin ligase Parkin in GH3 cells [94]. Furthermore, it was found that knocking down PINK1 significantly promotes the production of ROS, the increase in ATP, and cell apoptosis induced by T-2 toxin [104]. This indicates that PINK1-mediated mitophagy plays a protective role in T-2 toxin-induced oxidative stress damage and cell apoptosis. Additionally, Huang et al. demonstrated that knocking down the NFE2L2 gene significantly blocks the expression of PINK1 [94]. This indicates that T-2 toxin-induced PINK1-mediated mitophagy is partly dependent on the activation of the NFE2L2 pathway.

Wan et al. showed that an exposure to T-2 toxin significantly reduces the gene and protein expression related to glycolysis and the TCA cycle in GH3 cells. These include pyruvate kinase isozymes M1/M2 (PKM1/2), malate dehydrogenase (MDH), aconitate hydratase (Aco2), isocitrate dehydrogenase (NAD) subunit α (IDH), and ATP citrate lyase. This suggests that an exposure to T-2 toxin can damage mitochondrial energy metabolism.

Huang et al. reported that exposing GH3 cells (a rat pituitary cell line) to a high dose of T-2 toxin (i.e., 40 nM for 24 h) can decrease mitochondrial membrane potential, reduce the expression of the Bcl-2 protein and increase the expression of the Bax protein, ultimately resulting in cell apoptosis [94]. Similarly, Zhang et al. reported that exposing N2a neuronal cells to 5–80 ng/mL of T-2 toxin can cause a decrease in mitochondrial membrane potential and the expression of the Bcl-XL protein and an increase in the expression of Bax proteins and the activity of caspases-9 and -3, finally leading to cell apoptosis in a dose-dependent manner [12]. Previous studies have shown that the activation of p53 can activate Bax, which then makes the mitochondrial membrane permeable and triggers apoptosis [12,94]. It was also reported that an exposure to T-2 toxin can activate the expression of the p53 protein in neuronal cells [12]. This indicates that the activation of the p53 pathway also partly contributes to mitochondrial dysfunction caused by T-2 toxin.

Zhang et al. also found that treatment with T-2 toxin significantly increases the activity of caspase-8 and reduces the expression of the pro-BH3 interacting domain death agonist (BID) protein [12]. Similar findings were also observed in T-2 toxin-induced toxicity in GH3 cells. It is well known that caspase-8 is a biomarker of the extrinsic apoptotic pathway (i.e., the death receptor apoptosis pathway) [12]. Therefore, the activation of the extrinsic apoptotic pathway caused by T-2 toxin exposure may also contribute to its neurotoxicity.

**Figure 3:**
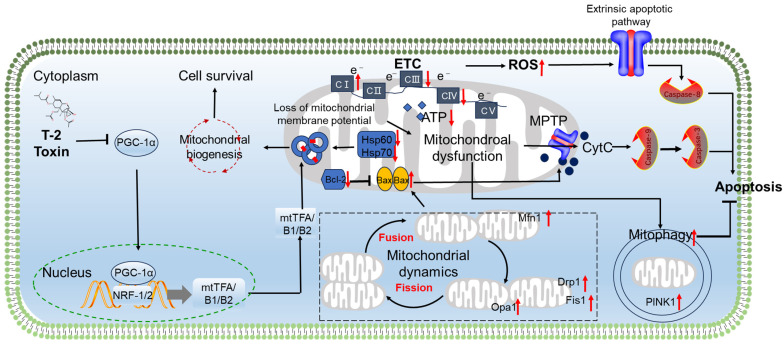
Schematic diagram of the proposal mechanisms for T-2 toxin-induced mitochondrial dysfunction and apoptosis in neuronal cells. T-2 toxin exposure can trigger mitochondrial impairment and apoptosis in neuronal cells. This mitochondrial dysfunction is linked to disruptions of mitochondrial biosynthesis, dynamics, mitophagy and electron transport chains. In detail, T-2 toxin exposure can inhibit the expression of PGC-1α, then inhibit the mitochondrial biogenesis via the inactivation of NRF-1/2 and mtTFA/B1/B2 signals. Mitochondrial dysfunction can induce both intrinsic (i.e., the release of CytC from damaged mitochondria triggers the activation of caspases-9 and -3) and extrinsic apoptotic (i.e., the activation of caspase-8) pathways, ultimately leading to cell death. ↑ indicates upregulation by T-2 toxin; ↓ indicates downregulation by T-2 toxin. → indicates promotion; 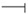 indicates inhibition.

Overall, T-2 toxin exposure has been shown to trigger mitochondrial impairment and apoptosis in neuronal cells. This mitochondrial dysfunction is linked to disruptions in key processes such as mitochondrial biosynthesis, dynamics, mitophagy and electron transport chains. The resulting dysregulation of mitochondrial function can activate both intrinsic and extrinsic apoptotic pathways, ultimately leading to cell death. As illustrated in Figure 3, these events involve multiple signaling pathways, including PGC1α, intrinsic apoptotic, extrinsic apoptotic, p53, NRF-2α, and PINK1 pathways.

### 5.3. Role of Inflammatory Responses and Cell Pyroptosis

T-2 toxin elicits inflammatory reactions in the central nervous system [106]. Nakajima et al. reported that maternal T-2 toxin exposure in mice markedly boosts astrocyte activation in cerebral cortex tissue, evidenced by elevated GFAP and STAT3 protein levels. This suggests that STAT3-mediated transcriptional GFAP upregulation in cerebellar astrocytes is essential in T-2 toxin-mediated neurotoxicity [107]. In murine models, T-2 toxin administration also significantly increased metallothionein (MT)-I/II-positive cells in brain regions like the dentate gyrus hilus, cerebral cortex, corpus callosum and cerebellum at doses of ≥3 mg/kg or 9 mg/kg body weight post-weaning, reflecting oxidative stress and inflammatory induction [107]. Previous investigations revealed that T-2 toxin concentrations from 0.625 ng/mL to 10 ng/mL provoke cytotoxicity, excessive ROS generation, oxidative stress, mitochondrial impairment, autophagy induction and mitochondrial apoptotic pathway activation in BV2 microglial cells, with effects depending on dose and duration. This underscores the vulnerability of microglia to T-2 toxin [72]. Similarly, Weidner et al. observed a substantial cellular toxicity and apoptosis in human primary astrocytes at low T-2 toxin exposures (at 1 nM to 10 μM) [36]. Microglial activation by T-2 toxin in mice leads to cognitive deficits, and suppressing this activation alleviates learning and memory dysfunction [37]. These results imply that astrocytes are the primary targets of T-2 toxin, and their activation is fundamental to neurotoxicity mechanisms. Li et al. established that microglia activation induced by T-2 toxin involves MAPK and NF-κB pathway stimulation in BV-2 cells [37]. NF-κB, a central inflammatory regulator, governs the transcription of pro-inflammatory genes like IL-1β, COX-2, iNOS, IL-6, and TNF-α [108]. In resting states, NF-κB is bound in the cytoplasm by IκB proteins. Upon stimulation by inflammatory cytokines, IκB degradation enables NF-κB nuclear translocation and gene transcription initiation. This mechanism is vital in microglia, the CNS’s primary immune cells, where NF-κB upregulation in pathological states can worsen disease via cellular death and inflammation amplification [109]. In another study, it was documented that T-2 toxin treatment at 5 ng/mL for 24 h elevates the phosphorylation of NF-κB and IκBα proteins, subsequently upregulating COX-2, CD11B, and mRNA levels for IL-1β, iNOS, IL-6, and TNF-α in BV2 cells [37].

Additionally, T-2 toxin exposure robustly activates the MAPK pathway in microglia. In treated BV2 cells, phosphorylated JNK and Erk proteins increase significantly, but not p-p38. The pharmacological inhibition of JNK and Erk pathways suppresses T-2 toxin-induced TNF-α, COX-2, IL-6, and IL-1β mRNA expression, indicating their contribution to neuroinflammatory responses. MAPK pathways serve as upstream regulators of NF-κB signaling [110], and T-2 toxin’s impact on JNK and Erk may influence NF-κB, though the molecular details require further exploration. T-2 toxin also upregulates BTG2 mRNA and protein expression in mouse hippocampus, cortex tissues, and BV2 cells [84]. Knocking down BTG2 attenuates T-2 toxin-induced neuroinflammation in BV2 cells, as seen by the reduced IL-1β, IL-6, and TNF-α expression, highlighting BTG2’s key role in microglial activation [84]. Moreover, inhibiting BTG2 expression reverses T-2 toxin-mediated PI3K/AKT and NF-κB pathway activation in microglia. PI3K/AKT pathway inhibition with LY294002 decreases p-IκB-α and p-NF-κB levels, lowering inflammatory markers IL-1β, IL-6, and TNF-α [84]. This evidence reveals BTG2’s involvement in microglial activation through PI3K/AKT/NF-κB signaling. BTG2 participates in diverse physiological and pathological functions, including cell differentiation, proliferation, apoptosis, and tumor suppression [111]. It is regulated by p53, suggesting that T-2 toxin-induced p53 activation might modulate inflammation via BTG2, but this complex mechanism needs additional study [111].

Liu et al. demonstrated that T-2 toxin at 5–80 nM over 1–12 h downregulated AT-hook transcription factor (AKNA) expression in GH3 cells dose- and time-dependently [112]. AKNA is crucial for immune response, inflammation, development, cancer, autoimmunity and neurogenesis [113]. As a master regulator of inflammation, AKNA silencing reduces T-2 toxin-induced inflammatory cytokines such as TNF-α, IL-1β, and IL-6, implying its role in neuroinflammatory outcomes [112,113,114]. T-2 toxin also increases phospho-PKA/CREB expression. Blocking PKA, NF-κB, p38, and Erk activities using gene silencing or inhibitors enhances AKNA expression in T-2 toxin-treated GH3 cells, indicating that PKA/CREB, NF-κB, and MAPK pathways negatively regulate AKNA to control inflammation [112].

Pyroptosis, a type of programmed cell death that generates intense inflammation, has been linked to inflammatory damage in the brain [115]. The assembly of inflammasomes elicits the activation of caspase proteins. These activated caspases then promote the formation of the N-terminal pore-forming gasdermin D (GSDMD) fragment (i.e., GSDMD-NT) [116]. Subsequently, IL-1β and IL-18 mature and are released through the ruptured cell membrane, leading to pyroptosis activation. Pei et al. discovered that, when mice were orally treated with T-2 toxin at a dose of 0.5–2 mg/kg body weight for 28 days, it significantly increased the expression of caspase-1, cleaved-IL-1β, IL-18, and GSDMD proteins in the brain tissues [117]. Most pyroptosis processes are initiated by the NLRP3 inflammasome complex [90]. The endogenous host sensor activated by pathogens initiates macrophage pyroptosis through the deubiquitylation of NLRP3, resulting in GSDMD cleavage and the leakage of cellular contents [90]. So, it follows that the NLRP3 inflammasome serves as an important mediator between extracellular stimulation and pyroptosis. In addition to the NLRP3 protein, the NLRP3 inflammasome also consists of the ASC recruiting domain and caspase-1 protein [118]. Similarly, in HT22 neuronal cells, an exposure to T-2 toxin can upregulate the expression of high-mobility group B1 protein (HMGB1), NLRP3, ASC, caspase-1 and GSDMD-NT proteins. The pharmacological inhibition of GSDMD activation by dimethyl fumarate or the silencing of HMGB1 gene expression by SiRNA can significantly ameliorate the T-2 toxin-induced activation of the NLRP3/caspase-1 inflammasome, then reduce cell pyroptosis [67]. This suggests that T-2 toxin exposure can activate cell pyroptosis in a GSDMD-dependent manner, which may also involve the activation of the NLRP3/caspase-1 inflammasome pathway and the HMGB1 pathway. NF-κB acts as a promoter molecule and translocates into the nucleus to elicit the activation of NLRP3 inflammasomes [67]. Currently, it remains unclear whether the activation of the NLRP3 inflammasome is due to the activation of NF-κB caused by T-2 toxin.

**Figure 4:**
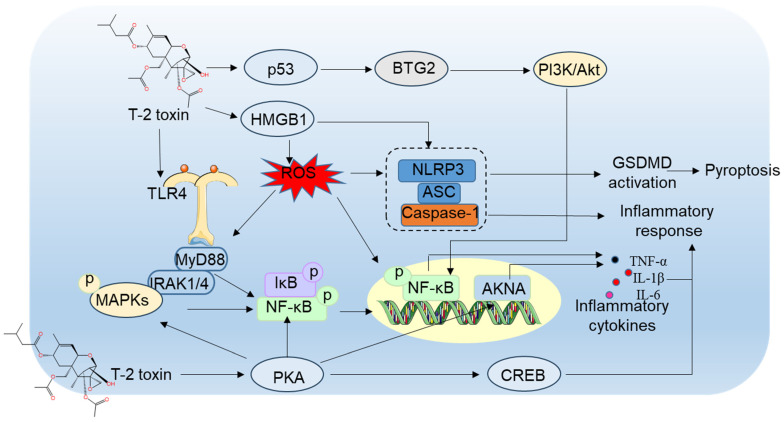
Schematic diagram of the proposed actional mechanisms for T-2 toxin-induced inflammatory responses and cell pyroptosis in neuronal or glial cells. T-2 toxin exposure can induce inflammatory responses in glial cells or neurons by activating the MAPK, AKNA, PKA/CREB, GSDMD, HMGB1, NF-κB and NLRP3 pathways. → indicates promotion.

The available research data suggest that T-2 toxin exposure can induce inflammatory responses in nerve tissues by activating the MAPK, AKNA, PKA/CREB, GSDMD, HMGB1, NF-κB and NLRP3 pathways (Figure 4). These findings offer crucial information for the targeted intervention of T-2 toxin-induced neuroinflammation and neurodegenerative diseases. The activation of these signaling pathways plays a pivotal role in the development of neuroinflammation, and understanding these mechanisms can help in formulating strategies to mitigate the harmful effects of T-2 toxin on the nervous system. Glial cells, which are known to be involved in neuroinflammatory processes, may also be affected by the activation of these pathways during T-2 toxin exposure. Further research is needed to fully understand the complex interplay between these signaling pathways, glial cells, and the overall process of T-2 toxin-induced neuroinflammation.

### 5.4. Role of Autophagy

Autophagy, a cellular degradation process, plays a crucial role in maintaining neuronal health by removing damaged organelles (including mitochondria and endoplasmic reticulum) and protein aggregates caused by DNA damage, hypoxia, nutrient deprivation, and oxidative stress [119]. Its dysfunction is increasingly recognized as a significant factor in the pathogenesis of various neurotoxicity or neurological diseases. It has been reported that the activation of autophagy is involved in the maintenance of neuronal homeostasis, particularly in response to oxidative stress and mitochondrial dysfunction induced by drugs and environmental toxins (such as colistin, methylmercury, and bupivacaine) [120,121,122]. In general, the fate of the cell depends on the interplay between pro-apoptotic factors and autophagy, wherein the latter blocks the induction of apoptosis or acts to delay apoptotic cell death; conversely, the activation of pro-apoptotic caspases shuts off autophagy. Notably, the formation of autophagosomes was observed using transmission electron microscopic examination in neuronal cells exposed to low doses of T-2 toxin [94]. Sun et al. reported that T-2 toxin treatment at 2.5–5 ng/mL significantly upregulated the expression of Beclin1 and LC3II proteins, promoted the formation of autophagosomes and autolysosomes and upregulated autophagy flux in BV2 cells [72]. Furthermore, Guo et al. demonstrated that the T-2 toxin-induced formation of autophagosomes and autolysosomes is partly dependent on the activation of autophagy-related gene 5 (ATG5) [104]. The inhibition of autophagy by chloroquine, a specific autophagy degradation inhibitor, significantly exacerbated T-2 toxin-induced caspase-3-dependent cell apoptosis [72]. This indicates that autophagy activation by T-2 toxin plays a protective role in the CNS.

A recent study showed that T-2 exposure in rats significantly downregulated the expression of ATG5 and mammalian target of rapamycin (mTOR) mRNAs on the 1st day, but significantly upregulated the expression of ATG5 and mTOR mRNA in brain tissue on the 3rd day post-T-2 exposure; the autophagy gene LC3B was significantly upregulated [49]. It is known that mTOR controls protein synthesis by activating S6 kinase 1 (S6K1) and 4E binding protein 1 and regulates autophagy via regulating the expression of ATG5 [123]. Consistently, Wu et al. showed that rapamycin, a special mTOR inhibitor, can markedly reduce T-2 toxin-induced apoptosis [94]. Additionally, T-2 toxin treatment also activated mitophagy, which was evident by the increased expression of mitophagy-specific proteins NIX, PINK1 and Parkin mRNA and proteins in GH3 cells, and the inhibition of PINK1-mediated mitophagy also promoted T-2 toxin-induced cell apoptosis.

In short, the current evidence indicates that T-2 toxin exposure can promote the formation of autophagosomes and autolysosomes and upregulate autophagy flux in nerve cells. It also induces mitophagy. These processes involve the mTOR, ATG5, and PINK1 pathways. Autophagy activation plays a protective role in T-2 toxin-induced cytotoxicity and apoptosis in nerve cells.

### 5.5. The Induction of Cell Cycle Arrest and Cellular Senescence

In neuronal cells, an exposure to T-2 toxin can induce cell cycle arrest and senescence [71,97,124]. For example, Agrawal et al. reported that exposing IMR-32 cells to 40 ng/mL of T-2 toxin for 8–24 h resulted in sub-G1 arrest and significantly elevated the mRNA levels of CDK2, CDK6, cyclin A and p21 [71]. Consistently, Fatima et al. discovered that treating GH3 cells with 40 nM of T-2 toxin significantly promoted G1 phase arrest, upregulated the expression of p16 and p21 proteins and downregulated the levels of cyclin D1, CDK4 and p-RB. Moreover, this process might be partly regulated by the p53 and MAPK pathways [71,97].

T-2 toxin treatment was demonstrated to induce cell cycle arrest and senescence in SH-SY5Y neuronal cells via upregulating the expression of p16, p21, and p53 and enhancing the activity of SA-β-gal [125]. Inhibiting HIF-α expression significantly decreased the expression of p53, p21 and p16 proteins; reduced the increased SA-β-gal activity; and ultimately alleviated T-2 toxin-induced neuronal cell cycle arrest and senescence. This implies that T-2 exposure-induced HIF-α activation partly contributes to cell cycle arrest and senescence in neuronal cells [125]. Additionally, T-2 toxin exposure can alter the expression of Alzheimer’s disease-related proteins, such as Tau, phosphorylated Tau (p-Tau), and amyloid precursor protein, indicating a significant association between T-2 toxin exposure and the development of neurodegenerative diseases [125].

### 5.6. Imbalance of Gut Microbiota

Research has shown that short-chain fatty acids (SCFAs) impact the cellular system and interact with gut–brain signaling pathways. Specifically, they can regulate immune, endocrine, neural, and humoral pathways, reduce inflammatory factors, activate the vagus nerve, and protect neurons against damage induced by environmental toxins via the gut–brain axis [126]. For example, Wu et al. reported that a dietary intake of 1 mg/kg T-2 toxin increased the enrichment of g_norank_f_T34, Faecalibacterium, and f_Clostridium_methylpentosum_group, while significantly reducing the levels of acetic acid, propionic acid, butyric acid and total SCFAs in the intestinal tissues of weaned piglets [127]. Su et al. showed that T-2 toxin exposure significantly increased the abundance of Enterobacteriaceae and decreased the abundance of Lactobacillus in the intestinal tissues of mice [128]. It was demonstrated that the increased Shigella abundance in the intestinal tissues induced by T-2 toxin is positively associated with inflammatory markers (e.g., TNF-α and IL-6) in the hippocampal tissues. Reversing these changes in gut microbiota can significantly improve T-2 toxin-induced neuroinflammation and neuro-memory dysfunction [128], indicating that the T-2 toxin-induced disruption of gut microbiota is involved in regulating neuroinflammatory responses and neurobehavioral disorders. Huang et al. reported that T-2 toxin disturbed the composition of gut microbiota, particularly Faecalibaculum and Allobaculum, which were positively correlated with anorexia and the levels of serum cholecystokinin, glucagon-like peptide-1, 5-HT, IL-1β, IL-6 and TNF-α [38]. These data suggest that T-2 toxin-induced changes in gut microbiota may partly contribute to its induction of neuroinflammation and anorexia. However, to date, the precise molecular mechanisms remain unclear, and more experimental evidence and further studies are needed.

## 6. Chemo-Protective Agents for T-2 Toxin-Induced Neurotoxicity

It was reported that several antioxidants, natural products and specific small-molecule inhibitors can effectively mitigate T-2 toxin-mediated neurotoxicity by simultaneously targeting oxidative stress, inflammatory cascades, and programmed cell death pathways (as shown in Table 2).

### 6.1. Antioxidants

GSH, a crucial tripeptide that regulates cellular detoxification, antioxidant defense, thiol homeostasis, and proliferation, is biosynthesized in the cytosol under strict control. Experimental research has repeatedly shown that T-2 exposure significantly depletes neuronal GSH reserves at both the tissue and cellular levels [12,74,88]. Due to the limited membrane permeability of exogenous GSH, oral supplementation is ineffective for direct intracellular delivery. NAC primarily increases GSH levels by providing the amino acid L-cysteine, which is the rate-limiting substrate for de novo GSH synthesis [129,130]. Zhang et al. reported that NAC supplementation can notably increase intracellular GSH levels, thereby significantly reducing T-2 toxin-induced caspase-mediated neuronal cell apoptosis [12]. Sun et al. found that NAC supplementation can markedly lower intracellular ROS levels and alleviate T-2 toxin-induced cytotoxicity in BV-2 cells [72]. Moreover, NAC supplementation can also significantly relieve T-2 toxin-induced cell pyroptosis by inhibiting the NLRP3 pathways [131], which may be attributed to its radical scavenging ability. Studies in animal models have indicated that NAC supplementation can effectively improve the clinical symptoms or pathological processes of neurodegenerative diseases or neurotoxicity induced by environmental toxins through the targeted inhibition of oxidative stress or inflammatory responses in neuronal cells [132,133,134,135,136]. This is in line with recent clinical trials that have shown that oral NAC supplementation can notably reduce neurotoxicity caused by chemotherapeutic agents (e.g., paclitaxel or oxaliplatin) in cancer patients [137,138].

Vitamin E, also known as alpha-tocopherol, is a lipid-soluble vitamin that serves as a potent antioxidant against oxidative damage. It was reported that vitamin E supplementation at 100 mg/kg body weight can significantly inhibit the production of ROS and MDA, upregulate the activities of GPX and GSH levels, and reduce the levels of IL-1β and TNF-α mRNAs in order to markedly attenuate T-2 toxin-induced mouse brain damage [74]. Notably, in the brain tissues, the transport of BBB is regulated by several transporters, such as α-tocopherol (binding) transport protein, phospholipid transfer protein, and scavenger receptor class B type 1, whose kinetics are quite slow in mammals [139]. This is the key reason for the lower levels of vitamin E in brain tissues compared to other organs (such as the liver) during dietary supplementation [139]. This a key limitation for vitamin E application in clinical practice.

In addition, several antioxidants such as melatonin, vitamin C, silymarin, selenium, L-carnitine, alpha-tocopherol, and coenzyme Q10 (CoQ10) have been demonstrated to have protective effects against T-2 toxin-induced cytotoxicity and tissue damage via scavenging radicals [127,140,141,142,143,144,145]. Clinical trials have also confirmed that supplementation with these antioxidants can effectively improve cognitive impairment and anorexia caused by anti-cancer drugs [146,147,148]. Therefore, it is worthwhile to further explore whether these antioxidants can effectively improve T-2 toxin-induced neurobehavioral defects or neurotoxicity, and more clinical trial studies are required.

### 6.2. Small-Molecule Inhibitors

Several small-molecule inhibitors, including PDTC, SP600125, PD98059, dimethyl fumarate, VX-765, YC-1, and LY294002, can target the NF-κB, JNK, Erk, GSDMD, caspase-1, HIF-1, and PI3K/Akt pathways, respectively, to inhibit neuroinflammatory responses, mitochondrial dysfunction, cell apoptosis, and pyroptosis caused by T-2 toxin exposure in neuronal or glial cells [37]. For instance, VX-765 and dimethyl fumarate can significantly inhibit the expression of caspase-1 and GSDMD-NT proteins in HT22 cells, respectively, thereby attenuating T-2 toxin-induced neuroinflammatory responses and neuronal pyroptosis [67]. Li et al. demonstrated that PDTC, SP600125, and PD98059 can markedly inhibit the NF-κB, JNK, and Erk pathways, respectively, and subsequently reduce microglial activation, as evidenced by the decreased expression of TNF-α, IL-6, COX-2, and IL-1β mRNA in BV-2 cells [37]. Zhao et al. reported that YC-1 supplementation can effectively inhibit the upregulation of HIF-1α induced by T-2 toxin. It then significantly downregulates intracellular MDA and ROS levels, while upregulating the expression of APP and p-Tau proteins in BV-2 cells via activating the JNK pathway. Inhibiting JNK can mitigate the adverse effects of YC-1 treatment, suggesting that HIF-1α may have a dual role, and co-targeting HIF-1α and JNK could be a new strategy to improve T-2 toxin-induced neurotoxicity [149]. Li et al. found that LY294002’s targeted inhibition of PI3K/Akt in BV-2 cells, then significantly inhibited the activation of NF-κB and the expression of its downstream genes, such as IL-1β, IL-6, and TNF-α mRNAs [84]. Additionally, 4-phenylbutyrate (4-PBA), an ER stress inhibitor, can effectively attenuate T-2 toxin-induced cytotoxicity and apoptosis in porcine renal epithelial cells [150] and goat endometrium epithelial cells [151]. Several clinical trials have shown that 4-PBA supplementation has potential therapeutic effects on inflammatory or neurological disorder-related diseases [152,153,154]. This information indicated that 4-PBA may be a promising candidate drug against T-2 toxin-induced neurotoxicity in animals and humans. Recently, Chen et al. reported that the local administration of AHN 1-055 hydrochloride, a dopamine transporter inhibitor, can significantly alleviate T-2 toxin-induced depression-like symptoms in mice [65]. Similarly, knocking down the dopamine transporter can produce obvious antidepressant like effects in a mouse model [155].

### 6.3. Natural Products

Plant natural products such as flavonoids, polysaccharides and peptides can improve various neurodegenerative diseases (e.g., cerebral ischemia, stroke, and Alzheimer’s disease) or neurotoxicity induced by environmental toxins or drugs [90,156,157]. These agents achieve this neuroprotective effect by alleviating oxidative stress, regulating cytokine levels and autophagy flow, and inhibiting excitotoxicity.

It has been reported that certain natural products such as saffron, daucosterol, resveratrol, quercetin, and betulinic acid have strong antioxidant, anti-inflammatory, and immune-regulatory activities by targeting NFE2L2, PGC-1α, NF-κB, NLRP3, and MAPK pathways. They exhibit potential protective effects against neurotoxicity caused by several mycotoxins [128,158,159,160]. For example, Gu et al. reported that the intraperitoneal injection of daucosterol (a phytosterol glycoside found in many plants) at 30 mg/kg body weight can significantly reduce damage to the BBB, hippocampal neuronal degeneration, and neuroinflammatory response in the brain tissues of mice exposed to T-2 toxin by specifically activating the PGC-1α protein [85]. Huang et al. reported that supplementing betulinic acid at 0.25–1 mg/kg body weight per day for fourteen days can significantly improve cognitive function, antioxidant capacity, and brain neurotransmitter (i.e., Ach, 5-HT, and DA) levels and inhibit apoptosis and the secretion of pro-inflammatory cytokines in brain tissues. It offers a protective effect against brain damage caused by T-2 toxin in a mouse model by specifically regulating NFE2L2, MAPK, and NF-κB pathways [74]. Moreover, it was shown that betulinic acid supplementation can significantly reduce T-2 toxin-induced cytotoxicity, hepatotoxicity, nephrotoxicity, enterotoxicity, spleen toxicity, and lymphotoxicity in vitro and in vivo by regulating the aforementioned pathways [161,162,163,164,165,166,167]. This indicates that betulinic acid supplementation can not only alleviate T-2 toxin-induced neurotoxicity but also systematically improve the toxic effects of T-2 toxin on different organs. Su et al. reported that an oral supplementation of resveratrol at 100 mg/kg can effectively reduce T-2 toxin-induced cognitive deficits, neuronal damage, and disruption of the gut–brain axis. The potential mechanisms may involve the restoration of Lactobacillus abundance and gut barrier integrity, thereby reduce systemic inflammation and restoring hippocampal synaptic protein expression and function in mice through gut–brain axis modulation [128]. A clinical trial further confirmed that resveratrol supplementation (up to 1 g orally twice daily) for 52 weeks can significantly treat or prevent neurodegenerative disorders in patients with Alzheimer’s disease [168,169]. These findings highlight the therapeutic potential of these compounds in dealing with oxidative stress- and inflammation-related neurotoxicity, providing promising directions for future research and clinical applications to diminish and prevent T-2 toxin-induced neurological disorders.

### 6.4. Other Neuroprotective Agents

Minocycline, a broad-spectrum tetracycline antibiotic, was recently found to exert broad protective effects in a multitude of neurological disorders, including spinal cord injury, stroke, multiple sclerosis, amyotrophic lateral sclerosis (ALS), Huntington’s disease, epilepsy, and PD, via inhibiting neuroinflammation [170,171,172].

It was reported that minocycline administration at 50 mg/kg body weight markedly reduced T-2 toxin exposure-induced microglia activation, downregulated the expression of inflammatory markers, including TNF-α, IL-1β, IL-6, COX-2, CD11b mRNAs or proteins in the brain tissues of mice, and finally improved learning, memory impairment, and locomotor inhibition in mice [37]. These findings highlight the utility of minocycline as a neuroprotective agent against T-2 toxin-induced neurotoxicity.

## 7. Conclusions and Future Perspectives

Neurotoxicity is a significant adverse effect resulting from exposure to T-2 toxin. Animal experiments have revealed that a continuous exposure to T-2 toxin allows the toxin to penetrate the BBB, then accumulate and damage CNS tissues, finally inducing a decline in learning, memory, cognition, and motor function. T-2 toxin-induced neurotoxicity is related to multiple detrimental cellular mechanisms, including oxidative stress, mitochondrial dysfunction, neuroinflammation, autophagy, ferroptosis and apoptosis. These cellular processes are in turn driven by signaling pathways, such as NFE2L2, NRF-2, PGC-1, p53, BTG2, AKNA, MAPK, Akt, mTOR, HMGB1, HIF-1, CREB, and NF-κB. T-2 toxin exposure can also disrupt the synthesis and release of various neurotransmitters, including DA, GABA, glutamate, norepinephrine, 5-HT and Ach. Additionally, T-2 toxin exposure can also induce the disruption of gut microbiota, which contributed to the induction of neuroinflammatory responses and neurobehavioral disorders. Accumulating evidence demonstrates that several specific antioxidants (such as NAC, vitamin E, vitamin C, silymarin, selenium, L-carnitine, alpha-tocopherol, and coenzyme Q10), natural products (such as betulinic acid, resveratrol, and daucosterol), small-molecule inhibitors (such as DTC, SP600125, PD98059, dimethyl fumarate, VX-765, YC-1, LY294002, and AHN 1–055 hydrochloride), and FDA-approved drugs (i.e., minocycline) can effectively mitigate T-2 toxin-mediated neurotoxicity via the inhibition of oxidative stress, the inactivation of inflammatory cascades, the reduction of programmed cell death pathways, and the reconstruction of gut microbiota. Notably, human clinical trials have suggested that supplementation with minocycline and resveratrol can improve both neuropathological injury and abnormal changes in neurobehavior. Minocycline and resveratrol may be considered as the promising candidate therapeutic drugs for T-2 toxin exposure-induced neurotoxicity in animals and humans, albeit more animal model evaluations and clinical studies are still needed.

To develop effective policy and regulatory implications for improving or treating T-2 toxin-induced neurotoxicity in humans and animals, several directions for future research are warranted:

(i) It is crucial to investigate the relationships between T-2 toxin at the environmental exposure dose and neurological disorders in animals and humans, as well as the underlying molecular mechanisms. Currently, there is still a dearth of evidence on the long-term health effects of T-2 toxin exposure in humans, especially for the nervous system and its contribution to the occurrence and the development of neurodegenerative diseases. We need to establish dose correlations between chronic low-level T-2 toxin exposure via diet and clinically relevant neurological disorders, such as anorexia, depression, and neurodegenerative diseases. Additionally, new knowledge relating to the underlying molecular mechanisms of T-2 toxin-induced neurotoxicity remains unclear. For example, miRNAs have been proven to play critical roles in brain injury and the development of neurological disorders, but their role in T-2 toxin-induced neurotoxicity is poorly understood. The application of emerging technologies such as single-cell sequencing, CRISPR-Cas9 and multi-omics may also help elucidate the underlying mechanisms.

(ii) The risk assessment of T-2 toxin exposure in human samples is too limited, and further research should be conducted in the future, which is of great significance for public health safety and risk assessment.

(iii) A co-exposure risk assessment for T-2 toxin with other environmental toxins should be especially noted. It has been reported that the combination exposure between T-2 toxin and NIV, HT-2 toxin or DON showed a marked synergistic toxic effect [173,174,175]. Therefore, it is necessary to assess the neurotoxic risk caused by the combined exposure of T-2 toxin and other coexisting environmental toxins, such as heavy metals or other toxins.

(iv) The precise molecular mechanisms by which the above-mentioned neuroprotectants operate against T-2 toxin-induced neurotoxicity remain largely unknown, especially their effects on the changes in various neurotransmitters. Additionally, human clinical trials and animal experiments are still required to fully substantiate the neuroprotection conferred by these protective agents. A synergistic policy framework combining preventive agriculture, technological innovation, global standardization and consumer empowerment is critical to curb T-2 toxin exposure.

## Figures and Tables

**Table 1:** Neurotoxic effects of T-2 toxin in vitro and in vivo.

Model		Treatment Time and Dosage	Toxic Effects and Potential Mechanisms	References
In vitro cell model	Mouse N2a cells	Cells were treated with T-2 toxin at the dose range of 5–80 ng/mL for 6–24 h	T-2 toxin can dose-dependently induce cytotoxicity and apoptosis. It involves the upregulation of oxidative stress and mitochondrial dysfunction. It also upregulated the expression of p53, Bax, and caspase-8 mRNAs and proteins, and downregulated the expression of NFE2L2 and HO-1 mRNAs and proteins.	[12]
	Rat PC12 cells	Cells were treated with T-2 toxin at 1–12 ng/mL for 24 h	T-2 toxin can dose-dependently induce cytotoxicity and apoptosis in PC12 cells. It also induced the production of ROS and a decrease in antioxidant enzymes’ activities, causing oxidative stress. Additionally, T-2 toxin treatment promoted NF-κB and HMGB1-mediated inflammation response.	[70]
	Human IMR-32 cells	Cells were treated with T-2 toxin at 10–100 ng/mL for 8–48 h	T-2 toxin treatment markedly induced oxidative stress, mitochondrial dysfunction, cell apoptosis, caspase activation, and cell cycle arrest. T-2 toxin treatment also upregulated the expression of p-ERK, p-JNK, p-p38, Ras, Raf, and c-Fos proteins. Targeting the inhibition of caspase, ERK, p38, and Raf can effectively inhibit the cell apoptosis caused by T-2 toxin treatment.	[71]
	Human astrocytes	Cells were treated with T-2 toxin at 1 nM–200 μM for 6–48 h	T-2 toxin treatment can dose-dependently induce cytotoxicity, apoptosis, and necrosis in human astrocytes.	[36]
	Mouse BV2 cells	Cells were treated with T-2 toxin at 1.25–5 ng/mL for 24 h	T-2 toxin treatment significantly induced ROS production, causing oxidative stress and mitochondrial dysfunction. Additionally, T-2 toxin significantly decreased the expression of NFE2L2 and HO-1 proteins, activated cell autophagy, and upregulated autophagy flux. Inhibition of autophagy promoted T-2 toxin-caused cytotoxicity and cell apoptosis.	[72]
	Mouse HT22 cells	HT22 cells were treated with T-2 toxin at 0.5–4 ng/mL for 1–24 h	T-2 toxin treatment dose-dependently induced a decrease in cell viability and cell apoptosis. It can also induce cell pyroptosis via triggering NLRP3-caspase-1 inflammasome and gasdermin D (GSDMD) pathways.	[67]
In vivo anima model	Male albino mice	T-2 toxin was single intravenously injected into mice at the doses of 2, 4, and 6 μg/kg body weight	T-2 toxin treatment caused gliosis in the cerebrum hippocampus tissues and acute inflammatory infiltrates at the focal areas, indicating encephalitis. T-2 toxin also damaged glial cells in the brain tissues and significantly downregulated the expression of aquaporin-4 mRNA.	[66]
	Male C57/BL6 mice	Mice were orally administered with T-2 toxin at doses of 0.5, 1, and 2 mg/kg body weight for 28 days	T-2 toxin can induce arrangement disorder in hippocampal cells and the abnormal staining of neurons. Additionally, it also induced neuronal apoptosis and NLRP3-caspase-1 inflammasome and GSDMD-mediated cell pyroptosis in hippocampal tissues.	[67]
	Male Kunming mice	Mice were orally administered with T-2 toxin at 4 mg/kg body weight for 14 days	Neuronal loss, cellular swelling, pericellular space widening, massive bleeding, and cognitive dysfunction were observed in T-2 toxin-treated mice. Additionally, apoptosis, inflammation, oxidative stress, and abnormal neurotransmitter levels in T-2 toxin-treated brain tissues were detected.	[74]
	Specific pathogen-free female Wistar rats	T-2 toxin treatment via oral administration at a signal dose of 2 mg/kg body weight	The main behavioral changes are manifested as psychological fear and poor mental state. Additionally, marked pathological damage was detected in the brains of the T-2 toxin-treated rats. Marked mitochondrial damage, autophagy activation, and apoptosis were also detected in the brain tissues.	[49]
	Swiss albino female mice	T-2 toxin treatment at 5.94 mg/kg body weight via the dermal route or at 1.54 mg/kg body weight via the subcutaneous route; mice were sacrificed at 1st, 3rd, and 7th days after exposure	A decrease in drinking water and weight loss was observed. T-2 toxin treatment through dermal or subcutaneous injection can induce ROS generation, GSH depletion, lipid peroxidation and the alteration of phase II detoxifying enzymes.	[61]
	Male C57BL6 mice	T-2 toxin treatment at 0.5–5 mg/kg body weight via oral administration	The appetite of mice is significantly suppressed after T-2 toxin exposure. It also increased the expression of IL-1β, TNF-α, and IL-6 mRNAs in the brain tissue and the expression of c-Fos protein in the brainstem tissues of mice after unilateral vagotomy.	[64]
	Pregnant Wistar rats	Rats were treated with a single oral dose of T-2 toxin at 2 mg/kg body weight	T-2 toxin induced cell apoptosis in fetal brains. T-2 toxin exposure also induced the expression of genes enriched in oxidative stress, mitogen-activated protein kinase (MAPK) (such as MEKK1 and c-jun), and other apoptosis-related genes (such as caspase-2 and insulin-like growth factor-binding protein-3-related genes).	[75]
	Female B6C3F1 mice	Mice were orally treated with T-2 toxin at 1 mg/kg body weight; mice were sacrificed at 0, 0.5, 2, 6 and 24 h after exposure to T-2 toxin	The behavior in food intake was markedly inhibited. T-2 toxin increased levels of plasma 5-HT and SP and resulted in anorexia in mice.	[76]
	Male Wistar rats	Rats were orally administrated with T-2 toxin at the doses of 0.2, 0.4 and 0.8 mg/kg body weight for 4 weeks	Decreased spatial orientation and learning efficiency and impaired memory function were observed. T-2 toxin exposure triggered marked hippocampal pathological damage. It also induced oxidative damage and cell apoptosis in hippocampal tissues.	[77]
	Male C57BL/6 J mice	Mice were orally administrated with 1.5 mg/kg T-2 toxin daily for 14 d	T-2 toxin induced a significant increment in immobile time in the tail suspension test and a decline in sucrose preference in the sucrose preference test. T-2 toxin treatment also significantly reduced the level of dopamine and elevated the expression of dopamine transporter protein in the reward center nucleus accumbens of the brain.	[65]

**Table 2:** A tabulated summary of potential protective agents targeting T-2 toxin-induced neurotoxicity in vitro and in vivo.

Models		Antioxidants/Natural Products/Small Molecular Inhibitors	Treatments	The Protective Effects	**Reference**
In vitro	N2a neuronal cells	NAC (an antioxidant)	Cells were treated with NAC at 5 mM or cotreated with T-2 toxin at 20 ng/mL for 24 h.	NAC supplementation significantly improved T-2 toxin exposure-induced GSH deletion, downregulated the activities of caspases-9 and -3, and finally attenuated T-2 toxin-induced cytotoxicity.	[12]
	Mouse microglia BV2 cells	NAC (an antioxidant)	Cells were pre-treated with NAC at 2.5 mM for 2 h, then co-treated with or without T-2 toxin at 2.5 ng/mL for 24 h.	NAC supplementation significantly improved T-2 toxin exposure-induced ROS production and cytotoxicity.	[72]
	Mouse hippocampal neuron cell line (HT22)	VX-765 (a caspase-1 inhibitor)	Cells were pretreated with dimethyl fumarate at 10 μM, then co-treated with or without T-2 toxin at 3 ng/mL for additional 24 h.	VX-765 pretreatment significantly attenuated T-2 toxin exposure-induced decreases in cell viability and pyroptosis, which was evident by the decreased expression of cleaved caspase-1, HMGB1, IL-1β, IL-18, GSDMD-NT proteins.	[67]
	Mouse microglia BV2 cells	PDTC (a NF-κB inhibitor), SP600125 (a JNK inhibitor), and PD98059 (an ERK inhibitor)	Cells were pre-treated with PDTC, SP600125, PD98059 for 2 h, then co-treated with T-2 toxin at 5 ng/mL for 12 h.	These inhibitors can all inhibit the T-2 toxin-induced expression of TNF-α, COX-2, IL-6, and IL-1β mRNAs in BV-2 cells, following to reduce microglial activation.	[37]
	Mouse microglia BV2 cells	LY294002 (a PI3K inhibitor)	Cells were pre-treated with LY294002 at 10 μM for 2 h, then co-treated with T-2 toxin at 5 ng/mL for 12 h.	LY294002 supplementation markedly inhibited the T-2 toxin exposure-induced activation of NF-κB and the expression of downstream inflammatory factors, such as TNF-α, IL-6, and IL-1β.	[84]
	Human neuroblastoma SH-SY5Y cells	YC-1 (a HIF-1α inhibitor)	Cells were pre-treated with YC-1 at 10 μM for 24 h, then co-treated with T-2 toxin at 6 nM for 2, 6, 12 or 24 h.	YC-1 treatment markedly inhibited T-2 toxin-induced cellular senescence by reducing the expression of p53, p21, p16, CCL-2, and IL-8 proteins.	[125]
	Mouse hippocampal neuron cell line (HT22)	Dimethyl fumarate (a NFE2L2 activator)	Cells were pretreated with dimethyl fumarate at 25 μM, then co-treated with or without T-2 toxin at 3 ng/mL for additional 24 h.	Dimethyl fumarate supplementation significantly attenuated the T-2 toxin exposure-induced release of LDH and the decreases in cell viability. It also reduced neuronal cell pyroptosis via inhibiting the expression of cleaved IL-1β, IL-18, and GSDMD-NT proteins.	[67]
In vivo	Male Kunming mice	Vitamin E (an antioxidant)	Mice were pretreated with vitamin E via the oral administration at 100 mg/kg body weight per day for 14 days, then mice were exposed with T-2 toxin by a single intraperitoneal injection of 4 mg/kg.	Vitamin E supplementation markedly attenuated T-2 toxin-induced oxidative stress and neuronal cell apoptosis by reducing the levels of ROS and MDA and increasing the GSH levels and GPX activities in the brain tissues of mice. It also decreased T-2 toxin-induced neuroinflammatory response.	[74]
	Mice	AHN 1–055 hydrochloride (a dopamine uptake inhibitor)	Mice were orally administration with T-2 toxin at 1.5 mg/kg body weight per day for 14 days. AHN 1–055 hydrochloride was infused into nucleus accumbens (NAc) of mice at 0.1 μg via cannulas before each behavioral test.	Pharmacological inhibition of DAT in NAc reverses the T-2 toxin-triggered depression-like behaviors and the reduced DA level in NAc in mice.	[65]
	Female Kunming mouse	Daucosterol (a natural product)	In animal model, mice were intraperitoneally injected with daucosterol at 30 mg/kg body, at 4 h before T-2 toxin treatment (at 1.57 mg/kg; subcutaneous injection).	Daucosterol supplementation markedly attenuated T-2 toxin-induced dysfunction of blood–brain barrier by promoting the transcription activation of PGC-1α and increasing the expression of claudin-5 (CLDN5), occludin (OCLN), and zonula occludens-1 (ZO-1) proteins in mouse brain tissues or human brain microvascular endothelial cells. It also reduced T-2 toxin exposure-induced neuroinflammatory response and neuronal cell apoptosis in the brain tissues of mice.	[85]
	Male C57BL/6 J mice	Resveratrol (a natural product)	Mice were given an intraperitoneal injection of 4 mg/kg T-2 toxin, then were given an intraperitoneal injection of 100 mg/kg resveratrol.	Resveratrol supplementation markedly ameliorated T-2 toxin exposure-induced spatial learning and memory impairments via upregulating the expression of postsynaptic density protein 95, synaptophysin I, and brain-derived neurotrophic factor (BDNF) proteins in the brain tissues. It can also restore intestinal flora disorders caused by T-2 toxin exposure and reduce the risk of inflammatory responses in the hippocampus, intestine, and the whole body.	[128]
	Mice	Betulinic acid (a natural product)	Mice were pretreated with betulinic acid via the oral administration at 0.25, 0.5, or 1 mg/kg body weight per day for 14 days, then were exposed with T-2 toxin through a single intraperitoneal injection of 4 mg/kg.	Betulinic acid supplementation markedly alleviated T-2 toxin exposure-caused cognitive impairment. It also markedly decreased T-2 toxin-induced oxidative stress and neuronal cell apoptosis by inhibiting the production of MDA and ROS and increasing the activities of GPX and GSH levels in brain tissues. Additionally, betulinic acid can reduce T-2 toxin-induced neuroinflammation and upregulate the levels of dopamine in the brain tissues.	[74]
	Mice	Minocycline (a semi-synthetic tetracycline antibiotic)	Mice were intraperitoneally injected with minocycline at 50 mg/kg body weight or co-treated intraperitoneally with or without T-2 toxin at 4 mg/kg body weight.	Minocycline supplementation markedly attenuated T-2 toxin exposure-caused spatial learning and memory and locomotor activity impairment via inhibiting microglial activation in the brain tissues of mice.	[37]

## Data Availability

No new data were created or analyzed in this study.

## References

[1] Ayelign A., De Saeger S. (2020). Mycotoxins in Ethiopia: Current status, implications to food safety and mitigation strategies. Food Control.

[2] Mukhtar K., Nabi B.G., Ansar S., Bhat Z.F., Aadil R.M., Mousavi Khaneghah A. (2023). Mycotoxins and consumers’ awareness: Recent progress and future challenges. Toxicon.

[3] Dai C., Tian E., Hao Z., Tang S., Wang Z., Sharma G., Jiang H., Shen J. (2022). Aflatoxin B1 Toxicity and Protective Effects of Curcumin: Molecular Mechanisms and Clinical Implications. Antioxidants.

[4] Dai C., Sharma G., Liu G., Shen J., Shao B., Hao Z. (2024). Therapeutic detoxification of quercetin for aflatoxin B1-related toxicity: Roles of oxidative stress, inflammation, and metabolic enzymes. Environ. Pollut..

[5] Dai C., Das Gupta S., Wang Z., Jiang H., Velkov T., Shen J. (2022). T-2 toxin and its cardiotoxicity: New insights on the molecular mechanisms and therapeutic implications. Food Chem. Toxicol..

[6] Mhlongo T.N., Ogola H.J.O., Selvarajan R., Sibanda T., Kamika I., Tekere M. (2020). Occurrence and diversity of waterborne fungi and associated mycotoxins in treated drinking water distribution system in South Africa: Implications on water quality and public health. Environ. Monit. Assess..

[7] Eskola M., Kos G., Elliott C.T., Hajšlová J., Mayar S., Krska R. (2020). Worldwide contamination of food-crops with mycotoxins: Validity of the widely cited ‘FAO estimate’ of 25. Crit. Rev. Food Sci. Nutr..

[8] Xu H., Wang L., Sun J., Wang L., Guo H., Ye Y., Sun X. (2022). Microbial detoxification of mycotoxins in food and feed. Crit. Rev. Food Sci. Nutr..

[9] Hao W., Guan S., Li A., Wang J., An G., Hofstetter U., Schatzmayr G. (2023). Mycotoxin Occurrence in Feeds and Raw Materials in China: A Five-Year Investigation. Toxins.

[10] Zain M.E. (2011). Impact of mycotoxins on humans and animals. J. Saudi Chem. Soc..

[11] Hussein H.S., Brasel J.M. (2001). Toxicity, metabolism, and impact of mycotoxins on humans and animals. Toxicology.

[12] Zhang X., Wang Y., Velkov T., Tang S., Dai C. (2018). T-2 toxin-induced toxicity in neuroblastoma-2a cells involves the generation of reactive oxygen, mitochondrial dysfunction and inhibition of Nrf2/HO-1 pathway. Food Chem. Toxicol..

[13] Omotayo O.P., Omotayo A.O., Mwanza M., Babalola O.O. (2019). Prevalence of Mycotoxins and Their Consequences on Human Health. Toxicol. Res..

[14] Yang C., Ning X., Wang B., Tian T., Chen Y., Ma L., Wang L. (2024). Association between spectrum of mycotoxins and semen quality: A cross-sectional study in Beijing, China. J. Hazard. Mater..

[15] Huybrechts I., Jacobs I., Biessy C., Aglago E.K., Jenab M., Claeys L., Zavadil J., Casagrande C., Nicolas G., Scelo G. (2024). Associations between dietary mycotoxins exposures and risk of hepatocellular carcinoma in a European cohort. PLoS ONE.

[16] Li D., Han J., Guo X., Qu C., Yu F., Wu X. (2016). The effects of T-2 toxin on the prevalence and development of Kashin-Beck disease in China: A meta-analysis and systematic review. Toxicol. Res..

[17] Jolly P.E., Shuaib F.M., Jiang Y., Preko P., Baidoo J., Stiles J.K., Wang J.S., Phillips T.D., Williams J.H. (2011). Association of high viral load and abnormal liver function with high aflatoxin B1-albumin adduct levels in HIV-positive Ghanaians: Preliminary observations. Food Addit. Contam. Part A Chem. Anal. Control Expo. Risk Assess..

[18] Desjardins A.E., Hohn T.M., McCormick S.P. (1993). Trichothecene biosynthesis in Fusarium species: Chemistry, genetics, and significance. Microbiol. Rev..

[19] Lei R.H., Jiang N., Zhang Q., Hu S.K., Dennis B.S., He S.S., Guo X. (2016). Prevalence of Selenium, T-2 Toxin, and Deoxynivalenol in Kashin-Beck Disease Areas in Qinghai Province, Northwest China. Biol. Trace Elem. Res..

[20] Sun L.Y., Li Q., Meng F.G., Fu Y., Zhao Z.J., Wang L.H. (2012). T-2 Toxin Contamination in Grains and Selenium Concentration in Drinking Water and Grains in Kaschin-Beck Disease Endemic Areas of Qinghai Province. Biol. Trace Elem. Res..

[21] Wang X.C., Liu X.D., Liu J.C., Wang G., Wan K.Y. (2012). Contamination Level of T-2 and HT-2 Toxin in Cereal Crops from Aba Area in Sichuan Province, China. Bull. Environ. Contam. Toxicol..

[22] Tan Y.F., Kuang Y., Zhao R.H., Chen B., Wu J.W. (2011). Determination of T-2 and HT-2 Toxins in Traditional Chinese Medicine Marketed in China by LC-ELSD after Sample Clean-Up by Two Solid-Phase Extractions. Chromatographia.

[23] Jiang T., Yan J., Tan H., Pu Z., Wang O., Liu T., Chen Z., Gao J., Wang J., Lin J. (2024). Prevalence of T-2 Toxin in the Food and Beverages of Residents Living in a Kashin-Beck-Disease Area of Qamdo, Tibet. Nutrients.

[24] Adhikari M., Negi B., Kaushik N., Adhikari A., Al-Khedhairy A.A., Kaushik N.K., Choi E.H. (2017). T-2 mycotoxin: Toxicological effects and decontamination strategies. Oncotarget.

[25] Weaver G.A., Kurtz H.J., Bates F.Y., Chi M.S., Mirocha C.J., Behrens J.C., Robison T.S. (1978). Acute and chronic toxicity of T-2 mycotoxin in swine. Vet. Rec..

[26] Fairhurst S., Marrs T.C., Parker H.C., Scawin J.W., Swanston D.W. (1987). Acute toxicity of T2 toxin in rats, mice, guinea pigs, and pigeons. Toxicology.

[27] Lin R., Sun Y., Ye W., Zheng T., Wen J., Deng Y. (2019). T-2 toxin inhibits the production of mucin via activating the IRE1/XBP1 pathway. Toxicology.

[28] Yang L., Yu Z., Hou J., Deng Y., Zhou Z., Zhao Z., Cui J. (2016). Toxicity and oxidative stress induced by T-2 toxin and HT-2 toxin in broilers and broiler hepatocytes. Food Chem. Toxicol..

[29] Li T., Sun W., Zhu S., He C., Chang T., Zhang J., Chen Y. (2024). T-2 Toxin-Mediated β-Arrestin-1 O-GlcNAcylation Exacerbates Glomerular Podocyte Injury via Regulating Histone Acetylation. Adv. Sci..

[30] Zhang Z., Xu Y., Wang J., Xie H., Sun X., Zhu X., Wei L., Liu Y. (2022). Protective Effect of Selenomethionine on T-2 Toxin-Induced Rabbit Immunotoxicity. Biol. Trace Elem. Res..

[31] Zhang Y.F., Yang J.Y., Meng X.P., Qiao X.L. (2019). l-arginine protects against T-2 toxin-induced male reproductive impairments in mice. Theriogenology.

[32] Janik-Karpinska E., Ceremuga M., Wieckowska M., Szyposzynska M., Niemcewicz M., Synowiec E., Sliwinski T., Bijak M. (2022). Direct T-2 Toxicity on Human Skin-Fibroblast Hs68 Cell Line-In Vitro Study. Int. J. Mol. Sci..

[33] Wang X., Wang Y., Qiu M., Sun L., Wang X., Li C., Xu D., Gooneratne R. (2017). Cytotoxicity of T-2 and modified T-2 toxins: Induction of JAK/STAT pathway in RAW264.7 cells by hepatopancreas and muscle extracts of shrimp fed with T-2 toxin. Toxicol. Res..

[34] Ravindran J., Agrawal M., Gupta N., Rao P.V. (2011). Alteration of blood brain barrier permeability by T-2 toxin: Role of MMP-9 and inflammatory cytokines. Toxicology.

[35] Weidner M., Huwel S., Ebert F., Schwerdtle T., Galla H.J., Humpf H.U. (2013). Influence of T-2 and HT-2 toxin on the blood-brain barrier in vitro: New experimental hints for neurotoxic effects. PLoS ONE.

[36] Weidner M., Lenczyk M., Schwerdt G., Gekle M., Humpf H.U. (2013). Neurotoxic potential and cellular uptake of T-2 toxin in human astrocytes in primary culture. Chem. Res. Toxicol..

[37] Li N., Yao C.Y., Diao J., Liu X.L., Tang E.J., Huang Q.S., Zhou Y.M., Hu Y.G., Li X.K., Long J.Y. (2023). The role of MAPK/NF-κB-associated microglial activation in T-2 toxin-induced mouse learning and memory impairment. Food Chem. Toxicol..

[38] Huang T., Li A., Zhang S., Fan J., Hua Z., Wang X., Zhang C., Yang X. (2024). The role of gut microbiota in anorexia induced by T-2 toxin. Ecotoxicol. Environ. Saf..

[39] Dai C., Xiao X., Sun F., Zhang Y., Hoyer D., Shen J., Tang S., Velkov T. (2019). T-2 toxin neurotoxicity: Role of oxidative stress and mitochondrial dysfunction. Arch. Toxicol..

[40] Li Y., Wang Z., Beier R.C., Shen J., De Smet D., De Saeger S., Zhang S. (2011). T-2 toxin, a trichothecene mycotoxin: Review of toxicity, metabolism, and analytical methods. J. Agric. Food Chem..

[41] Wu Q.H., Huang L.L., Liu Z.Y., Yao M., Wang Y.L., Dai M.H., Yuan Z.H. (2011). A comparison of hepatic in vitro metabolism of T-2 toxin in rats, pigs, chickens, and carp. Xenobiotica.

[42] Wu Q., Qin Z., Kuca K., You L., Zhao Y., Liu A., Musilek K., Chrienova Z., Nepovimova E., Oleksak P. (2020). An update on T-2 toxin and its modified forms: Metabolism, immunotoxicity mechanism, and human exposure assessment. Arch. Toxicol..

[43] Yang S., Van Poucke C., Wang Z., Zhang S., De Saeger S., De Boevre M. (2017). Metabolic profile of the masked mycotoxin T-2 toxin-3-glucoside in rats (in vitro and in vivo) and humans (in vitro). World Mycotoxin J..

[44] Yang S.P., Li Y.S., Cao X.P., Hu D.F., Wang Z.H., Wang Y., Shen J.Z., Zhang S.X. (2013). Metabolic Pathways of T-2 Toxin in in Vivo and in Vitro Systems of Wistar Rats. J. Agric. Food Chem..

[45] Wu Q., Dohnal V., Huang L., Kuca K., Yuan Z. (2010). Metabolic pathways of trichothecenes. Drug Metab. Rev..

[46] Sintov A., Bialer M., Yagen B. (1986). Pharmacokinetics of T-2-Toxin and Its Metabolite Ht-2-Toxin, after Intravenous Administration in Dogs. Drug Metab. Dispos..

[47] Sintov A., Bialer M., Yagen B. (1988). Pharmacokinetics and protein binding of trichothecene mycotoxins, T-2 toxin and HT-2 toxin, in dogs. Toxicon.

[48] Sintov A., Bialer M., Yagen B. (1987). Pharmacokinetics of T-2 tetraol, a urinary metabolite of the trichothecene mycotoxin, T-2 toxin, in dog. Xenobiotica.

[49] Guo P., Liu A., Huang D., Wu Q., Fatima Z., Tao Y., Cheng G., Wang X., Yuan Z. (2018). Brain damage and neurological symptoms induced by T-2 toxin in rat brain. Toxicol. Lett..

[50] Wu Q.H., Wang X., Yang W., Nussler A., Xiong L.Y., Kuca K., Dohnal V., Zhang X.J., Yuan Z.H. (2014). Oxidative stress-mediated cytotoxicity and metabolism of T-2 toxin and deoxynivalenol in animals and humans: An update. Arch. Toxicol..

[51] Dauchy S., Dutheil F., Weaver R.J., Chassoux F., Daumas-Duport C., Couraud P.O., Scherrmann J.M., De Waziers I., Declèves X. (2008). ABC transporters, cytochromes P450 and their main transcription factors: Expression at the human blood-brain barrier. J. Neurochem..

[52] Granberg L., Ostergren A., Brandt I., Brittebo E.B. (2003). CYP1A1 and CYP1B1 in blood-brain interfaces: CYP1A1-dependent bioactivation of 7,12-dimethylbenz(a)anthracene in endothelial cells. Drug Metab. Dispos..

[53] Boussadia B., Ghosh C., Plaud C., Pascussi J.M., de Bock F., Rousset M.C., Janigro D., Marchi N. (2014). Effect of status epilepticus and antiepileptic drugs on CYP2E1 brain expression. Neuroscience.

[54] Ghosh C., Hossain M., Solanki J., Dadas A., Marchi N., Janigro D. (2016). Pathophysiological implications of neurovascular P450 in brain disorders. Drug Discov. Today.

[55] Ye W., Lin R., Chen X., Chen J., Chen R., Xie X., Deng Y., Wen J. (2019). T-2 toxin upregulates the expression of human cytochrome P450 1A1 (CYP1A1) by enhancing NRF1 and Sp1 interaction. Toxicol. Lett..

[56] Narváez A., Izzo L., Pallarés N., Castaldo L., Rodríguez-Carrasco Y., Ritieni A. (2021). Human Biomonitoring of T-2 Toxin, T-2 Toxin-3-Glucoside and Their Metabolites in Urine through High-Resolution Mass Spectrometry. Toxins.

[57] Yang S., De Boevre M., Zhang H., De Ruyck K., Sun F., Zhang J., Jin Y., Li Y., Wang Z., Zhang S. (2017). Metabolism of T-2 Toxin in Farm Animals and Human In Vitro and in Chickens In Vivo Using Ultra High-Performance Liquid Chromatography- Quadrupole/Time-of-Flight Hybrid Mass Spectrometry Along with Online Hydrogen/Deuterium Exchange Technique. J. Agric. Food Chem..

[58] Arcella D., Gergelova P., Innocenti M.L., Steinkellner H. (2017). Human and animal dietary exposure to T-2 and HT-2 toxin. EFSA J. Eur. Food Saf. Auth..

[59] Arce-López B., Alvarez-Erviti L., De Santis B., Izco M., López-Calvo S., Marzo-Sola M.E., Debegnach F., Lizarraga E., López de Cerain A., González-Peñas E. (2021). Biomonitoring of Mycotoxins in Plasma of Patients with Alzheimer’s and Parkinson’s Disease. Toxins.

[60] Zhang J., You L., Wu W., Wang X., Chrienova Z., Nepovimova E., Wu Q., Kuca K. (2020). The neurotoxicity of trichothecenes T-2 toxin and deoxynivalenol (DON): Current status and future perspectives. Food Chem. Toxicol..

[61] Chaudhary M., Rao P.V. (2010). Brain oxidative stress after dermal and subcutaneous exposure of T-2 toxin in mice. Food Chem. Toxicol..

[62] Xiao H. (2023). Study on the Effects and Mechanisms of T-2 Toxin-Induced Damage to the Cerebral Cortex in Mice. Master’s Thesis.

[63] Zhang J., Zhang H., Liu S.L., Wu W.D., Zhang H.B. (2018). Comparison of Anorectic Potencies of Type A Trichothecenes T-2 Toxin, HT-2 Toxin, Diacetoxyscirpenol, and Neosolaniol. Toxins.

[64] Gaigé S., Djelloul M., Tardivel C., Airault C., Félix B., Jean A., Lebrun B., Troadec J.D., Dallaporta M. (2014). Modification of energy balance induced by the food contaminant T-2 toxin: A multimodal gut-to-brain connection. Brain Behav. Immun..

[65] Chen Z., Duan S., Li J., Su J., Lei H. (2024). T-2 toxin triggers depression-like behaviors via upregulation of dopamine transporter in nucleus accumbens of male mice. Ecotoxicol. Environ. Saf..

[66] Maroli N., Kalagatur N.K., Bhasuran B., Jayakrishnan A., Manoharan R.R., Kolandaivel P., Natarajan J., Kadirvelu K. (2019). Molecular Mechanism of T-2 Toxin-Induced Cerebral Edema by Aquaporin-4 Blocking and Permeation. J. Chem. Inf. Model..

[67] Pei X., Ma S., Hong L., Zuo Z., Xu G., Chen C., Shen Y., Liu D., Li C., Li D. (2025). Molecular insights of T-2 toxin exposure-induced neurotoxicity and the neuroprotective effect of dimethyl fumarate. Food Chem. Toxicol..

[68] Yarom R., Yagen B. (1986). T-2 toxin effect on the ultrastructure of myocardial microvasculature. Br. J. Exp. Pathol..

[69] Dai C., Hao Z., Shen J. (2025). The Hazards of Mycotoxins and Key Issues for Future Research. J. Food Saf. Food Qual..

[70] Pei X., Jiang H., Liu X., Li L., Li C., Xiao X., Li D., Tang S. (2021). Targeting HMGB1 inhibits T-2 toxin-induced neurotoxicity via regulation of oxidative stress, neuroinflammation and neuronal apoptosis. Food Chem. Toxicol..

[71] Agrawal M., Bhaskar A.S., Lakshmana Rao P.V. (2015). Involvement of mitogen-activated protein kinase pathway in T-2 toxin-induced cell cycle alteration and apoptosis in human neuroblastoma cells. Mol. Neurobiol..

[72] Sun T., Zhang Q., Li M., Tang S., Dai C. (2022). T-2 Toxin Induces Apoptotic Cell Death and Protective Autophagy in Mouse Microglia BV2 Cells. J. Fungi.

[73] Wang Y., Wang B., Wang P., Hua Z., Zhang S., Wang X., Yang X., Zhang C. (2024). Review of neurotoxicity of T-2 toxin. Mycotoxin Res..

[74] Huang Y., Zhu Z., Luo C., Ma C., Zhu L., Kong L., Li R., Wu J., Yuan Z., Yi J. (2022). Betulinic acid attenuates cognitive dysfunction, oxidative stress, and inflammation in a model of T-2 toxin-induced brain damage. Environ. Sci. Pollut. Res. Int..

[75] Sehata S., Kiyosawa N., Makino T., Atsumi F., Ito K., Yamoto T., Teranishi M., Baba Y., Uetsuka K., Nakayama H. (2004). Morphological and microarray analysis of T-2 toxin-induced rat fetal brain lesion. Food Chem. Toxicol..

[76] Sheng K., Lu X., Yue J., Gu W., Gu C., Zhang H., Wu W. (2019). Role of neurotransmitters 5-hydroxytryptamine and substance P in anorexia induction following oral exposure to the trichothecene T-2 toxin. Food Chem. Toxicol..

[77] Zhou J., Meng F., Li Q., Wang H., Zou N. (2026). Subchronic exposure to T-2 toxin triggered neurobehavioral damage in developing juvenile rats was associated with oxidative stress and mitochondrial pathway-induced apoptosis of hippocampal neurons. Chem. Biol. Interact..

[78] Chi M.S., El-Halawani M.E., Waibel P.E., Mirocha C.J. (1981). Effects of T-2 toxin on brain catecholamines and selected blood components in growing chickens. Poult. Sci..

[79] Wang J., Fitzpatrick D.W., Wilson J.R. (1998). Effects of the trichothecene mycotoxin T-2 toxin on neurotransmitters and metabolites in discrete areas of the rat brain. Food Chem. Toxicol..

[80] Navakkode S., Korte M. (2012). Cooperation between cholinergic and glutamatergic receptors are essential to induce BDNF-dependent long-lasting memory storage. Hippocampus.

[81] Tanaka T., Abe H., Kimura M., Onda N., Mizukami S., Yoshida T., Shibutani M. (2016). Developmental exposure to T-2 toxin reversibly affects postnatal hippocampal neurogenesis and reduces neural stem cells and progenitor cells in mice. Arch. Toxicol..

[82] Gasiorowska A., Wydrych M., Drapich P., Zadrozny M., Steczkowska M., Niewiadomski W., Niewiadomska G. (2021). The Biology and Pathobiology of Glutamatergic, Cholinergic, and Dopaminergic Signaling in the Aging Brain. Front. Aging Neurosci..

[83] Pang Y., Zhang L., Liu Q., Peng H., He J., Jin H., Su X., Zhao J., Guo J. (2022). NRF2/PGC-1α-mediated mitochondrial biogenesis contributes to T-2 toxin-induced toxicity in human neuroblastoma SH-SY5Y cells. Toxicol. Appl. Pharmacol..

[84] Li X., Long J., Yao C., Liu X., Li N., Zhou Y., Li D., Xiong G., Wang K., Hao Y. (2024). The role of BTG2/PI3K/AKT pathway-mediated microglial activation in T-2 toxin-induced neurotoxicity. Toxicol. Lett..

[85] Guo P., Lu Q., Hu S., Yang Y., Wang X., Yang X., Wang X. (2023). Daucosterol confers protection against T-2 toxin induced blood-brain barrier toxicity through the PGC-1α-mediated defensive response in vitro and in vivo. J. Hazard. Mater..

[86] Jomova K., Raptova R., Alomar S.Y., Alwasel S.H., Nepovimova E., Kuca K., Valko M. (2023). Reactive oxygen species, toxicity, oxidative stress, and antioxidants: Chronic diseases and aging. Arch. Toxicol..

[87] Lingappan K. (2018). NF-κB in Oxidative Stress. Curr. Opin. Toxicol..

[88] Lu Q., Zhu Y., Wang L., Mei M., Qiu Y., Liu Y., Fu S., Xiong J., Guo P., Wu Z. (2024). Peroxiredoxin 4 Ameliorates T-2 Toxin-Induced Growth Retardation in GH3 Cells by Inhibiting Oxidative Stress and Apoptosis. Molecules.

[89] Liang X., Yan Z., Ma W., Qian Y., Zou X., Cui Y., Liu J., Meng Y. (2020). Peroxiredoxin 4 protects against ovarian ageing by ameliorating D-galactose-induced oxidative damage in mice. Cell Death Dis..

[90] Peng X., Zhang X., Sharma G., Dai C. (2024). Thymol as a Potential Neuroprotective Agent: Mechanisms, Efficacy, and Future Prospects. J. Agric. Food Chem..

[91] Zhang X., Liu Y., Shen Z., Wang S., Wu C., Liu D., Tang S., Dai C. (2023). Osthole ameliorates myonecrosis caused by Clostridium perfringens type A infection in mice. One Health Adv..

[92] Liu Y., Chen C., Hao Z., Shen J., Tang S., Dai C. (2024). Ellagic Acid Reduces Cadmium Exposure-Induced Apoptosis in HT22 Cells via Inhibiting Oxidative Stress and Mitochondrial Dysfunction and Activating Nrf2/HO-1 Pathway. Antioxidants.

[93] Zhang X., Liu Y., Liu M., Ma Q., Hao Z., Tang S., Dai C. (2024). Ellagic acid supplementation ameliorates cisplatin-induced liver injury in mice by inhibiting the NF-κB pathway and activating the Nrf2/HO-1 pathway. One Health Adv..

[94] Huang D.Y., Cui L.Q., Liu X.L., Guo P., Lu Q.R., Wang X., Yuan Z.H. (2018). Protective mechanisms involving enhanced mitochondrial functions and mitophagy against T-2 toxin-induced toxicities in GH3 cells. Toxicol. Lett..

[95] Poprac P., Jomova K., Simunkova M., Kollar V., Rhodes C.J., Valko M. (2017). Targeting Free Radicals in Oxidative Stress-Related Human Diseases. Trends Pharmacol. Sci..

[96] Schieber M., Chandel N.S. (2014). ROS function in redox signaling and oxidative stress. Curr. Biol. CB.

[97] Fatima Z., Guo P., Huang D.Y., Lu Q.R., Wu Q.H., Dai M.H., Cheng G.Y., Peng D.P., Tao Y.F., Ayub M. (2018). The critical role of p16/Rb pathway in the inhibition of GH3 cell cycle induced by T-2 toxin. Toxicology.

[98] Bin-Umer M.A., McLaughlin J.E., Basu D., McCormick S., Tumer N.E. (2011). Trichothecene Mycotoxins Inhibit Mitochondrial Translation-Implication for the Mechanism of Toxicity. Toxins.

[99] Wan D., Wang X., Wu Q.H., Lin P.P., Pan Y.H., Sattar A., Huang L.L., Ahmad I., Zhang Y.Y., Yuan Z.H. (2015). Integrated Transcriptional and Proteomic Analysis of Growth Hormone Suppression Mediated by Trichothecene T-2 Toxin in Rat GH3 Cells. Toxicol. Sci..

[100] Jovaisaite V., Mouchiroud L., Auwerx J. (2014). The mitochondrial unfolded protein response, a conserved stress response pathway with implications in health and disease. J. Exp. Biol..

[101] Azem A., Oppliger W., Lustig A., Jeno P., Feifel B., Schatz G., Horst M. (1997). The mitochondrial hsp70 chaperone system—Effect of adenine nucleotides, peptide substrate, and mGrpE on the oligomeric state of mhsp70. J. Biol. Chem..

[102] Leu J.I.J., Barnoud T., Zhang G., Tian T., Wei Z., Herlyn M., Murphy M.E., George D.L. (2017). Inhibition of stress-inducible HSP70 impairs mitochondrial proteostasis and function. Oncotarget.

[103] Fang H.Q., Cong L.Z., Zhi Y., Xu H.B., Jia X.D., Peng S.Q. (2016). T-2 toxin inhibits murine ES cells cardiac differentiation and mitochondrial biogenesis by ROS and p-38 MAPK-mediated pathway. Toxicol. Lett..

[104] Guo J., Ye X., Zhao Y., Huang D., Wu Q., Ihsan A., Wang X. (2023). NRF-2α and mitophagy underlie enhanced mitochondrial functions and biogenesis induced by T-2 toxin in GH3 cells. Food Chem. Toxicol..

[105] Tábara L.-C., Segawa M., Prudent J. (2025). Molecular mechanisms of mitochondrial dynamics. Nat. Rev. Mol. Cell Biol..

[106] Mehrzad J., Malvandi A.M., Alipour M., Hosseinkhani S. (2017). Environmentally relevant level of aflatoxin B(1) elicits toxic pro-inflammatory response in murine CNS-derived cells. Toxicol. Lett..

[107] Nakajima K., Tanaka T., Masubuchi Y., Ito Y., Kikuchi S., Woo G.H., Yoshida T., Shibutani M. (2019). Developmental Exposure of Mice to T-2 Toxin Increases Astrocytes and Hippocampal Neural Stem Cells Expressing Metallothionein. Neurotox. Res..

[108] Anilkumar S., Wright-Jin E. (2024). NF-κB as an Inducible Regulator of Inflammation in the Central Nervous System. Cells.

[109] Gasparini C., Feldmann M. (2012). NF-κB as a target for modulating inflammatory responses. Curr. Pharm. Des..

[110] Ma X., Dang C., Kang H., Dai Z., Lin S., Guan H., Liu X., Wang X., Hui W. (2015). Saikosaponin-D reduces cisplatin-induced nephrotoxicity by repressing ROS-mediated activation of MAPK and NF-κB signalling pathways. Int. Immunopharmacol..

[111] Mao B., Zhang Z., Wang G. (2015). BTG2: A rising star of tumor suppressors (review). Int. J. Oncol..

[112] Liu X., Huang D., Guo P., Wu Q., Dai M., Cheng G., Hao H., Xie S., Yuan Z., Wang X. (2017). PKA/CREB and NF-κB pathway regulates AKNA transcription: A novel insight into T-2 toxin-induced inflammation and GH deficiency in GH3 cells. Toxicology.

[113] Ramírez-González A., Manzo-Merino J., Contreras-Ochoa C.O., Bahena-Román M., Aguilar-Villaseñor J.M., Lagunas-Martínez A., Rosenstein Y., Madrid Marina V., Torres-Poveda K. (2021). Functional Role of AKNA: A Scoping Review. Biomolecules.

[114] Ma W., Ortiz-Quintero B., Rangel R., McKeller M.R., Herrera-Rodriguez S., Castillo E.F., Schluns K.S., Hall M., Zhang H., Suh W.K. (2011). Coordinate activation of inflammatory gene networks, alveolar destruction and neonatal death in AKNA deficient mice. Cell Res..

[115] Fernandes-Alnemri T., Wu J., Yu J.W., Datta P., Miller B., Jankowski W., Rosenberg S., Zhang J., Alnemri E.S. (2007). The pyroptosome: A supramolecular assembly of ASC dimers mediating inflammatory cell death via caspase-1 activation. Cell Death Differ..

[116] Sun Y.B., Zhao H., Mu D.L., Zhang W., Cui J., Wu L., Alam A., Wang D.X., Ma D. (2019). Dexmedetomidine inhibits astrocyte pyroptosis and subsequently protects the brain in in vitro and in vivo models of sepsis. Cell Death Dis..

[117] Bergsbaken T., Fink S.L., Cookson B.T. (2009). Pyroptosis: Host cell death and inflammation. Nat. Rev. Microbiol..

[118] Garib F.Y., Rizopulu A.P., Kuchmiy A.A., Garib V.F. (2016). Inactivation of Inflammasomes by Pathogens Regulates Inflammation. Biochem. Biokhimiia.

[119] Tang D.L., Kang R., Vanden Berghe T., Vandenabeele P., Kroemer G. (2019). The molecular machinery of regulated cell death. Cell Res..

[120] Wang S.L., Xia B., Qiao Z.L., Duan L., Wang G.M., Meng W.J., Liu Z.F., Wang Y., Zhang M.Y. (2019). Tetramethylpyrazine attenuated bupivacaine-induced neurotoxicity in SH-SY5Y cells through regulating apoptosis, autophagy and oxidative damage. Drug Des. Dev. Ther..

[121] Gao Q., Jiang T., Zhao H.R., Wu L., Tian Y.Y., Ou Z., Zhang L., Pan Y., Lu J., Zhang Y.D. (2016). Activation of Autophagy Contributes to the Angiotensin II-Triggered Apoptosis in a Dopaminergic Neuronal Cell Line. Mol. Neurobiol..

[122] Dai C.S., Tang S.S., Velkov T., Xiao X.L. (2016). Colistin-Induced Apoptosis of Neuroblastoma-2a Cells Involves the Generation of Reactive Oxygen Species, Mitochondrial Dysfunction, and Autophagy. Mol. Neurobiol..

[123] Yoon M.S. (2017). mTOR as a Key Regulator in Maintaining Skeletal Muscle Mass. Front. Physiol..

[124] Pei X., Zhang W., Jiang H., Liu D., Liu X., Li L., Li C., Xiao X., Tang S., Li D. (2021). Food-Origin Mycotoxin-Induced Neurotoxicity: Intend to Break the Rules of Neuroglia Cells. Oxidative Med. Cell. Longev..

[125] Wang Y., Wang X., Wu Q. (2025). T-2 toxin induces senescence in human neuroblastoma SH-SY5Y cells by regulating the HIF-1α/p53/p21 pathway. Ecotoxicol. Environ. Saf..

[126] Dalile B., Van Oudenhove L., Vervliet B., Verbeke K. (2019). The role of short-chain fatty acids in microbiota-gut-brain communication. Nat. Rev. Gastroenterol. Hepatol..

[127] Wu Y., Xiao W., Xiao B., Wang Y., Li Y., Wu A., Zhang Q., Liu X., Liu S., Yuan Z. (2025). Melatonin Alleviates T-2 Toxin-Induced Intestinal Injury by Enhancing Gut Barrier Function and Modulating Microbiota in Weaned Piglets. J. Agric. Food Chem..

[128] Su S.Q., Xiong G.Y., Yao C.Y., Liu X.L., Xia Y.Y., Long J.Y., Li X.K., Wang L.M., Yi L., Xu W.W. (2025). T-2 toxin-induced spatial learning and memory impairment in mice via the gut-brain axis and the intervening effects of resveratrol. Ecotoxicol. Environ. Saf..

[129] Atkuri K.R., Mantovani J.J., Herzenberg L.A., Herzenberg L.A. (2007). N-Acetylcysteine--a safe antidote for cysteine/glutathione deficiency. Curr. Opin. Pharmacol..

[130] Jenkins D.D., Wiest D.B., Mulvihill D.M., Hlavacek A.M., Majstoravich S.J., Brown T.R., Taylor J.J., Buckley J.R., Turner R.P., Rollins L.G. (2016). Fetal and Neonatal Effects of N-Acetylcysteine When Used for Neuroprotection in Maternal Chorioamnionitis. J. Pediatr..

[131] Yang X., Liu P., Cui Y., Song M., Zhang X., Zhang C., Jiang Y., Li Y. (2022). T-2 Toxin Caused Mice Testicular Inflammation Injury via ROS-Mediated NLRP3 Inflammasome Activation. J. Agric. Food Chem..

[132] Bai H., Chen H., Du S., Qiu D., Li S., Ma T., Gao R., Zhang Z. (2025). N-Acetylcysteine Mitigates Ketamine Neurotoxicity in Young Rats by Modulating ROS-Mediated Pyroptosis and Ferroptosis. Mol. Neurobiol..

[133] Chen Y., Nan Y., Xu L., Dai A., Orteg R.M.M., Ma M., Zeng Y., Li J. (2025). Polystyrene nanoplastics exposure induces cognitive impairment in mice via induction of oxidative stress and ERK/MAPK-mediated neuronal cuproptosis. Part. Fibre Toxicol..

[134] Zhao P., Yuan Q., Liang C., Ma Y., Zhu X., Hao X., Li X., Shi J., Fu Q., Fan H. (2024). GPX4 degradation contributes to fluoride-induced neuronal ferroptosis and cognitive impairment via mtROS-chaperone-mediated autophagy. Sci. Total Environ..

[135] Xiong G., Zhao L., Yan M., Wang X., Zhou Z., Chang X. (2019). N-acetylcysteine alleviated paraquat-induced mitochondrial fragmentation and autophagy in primary murine neural progenitor cells. J. Appl. Toxicol..

[136] Park S.W., Kim S.H., Park K.H., Kim S.D., Kim J.Y., Baek S.Y., Chung B.S., Kang C.D. (2004). Preventive effect of antioxidants in MPTP-induced mouse model of Parkinson’s disease. Neurosci. Lett..

[137] Khalefa H.G., Shawki M.A., Aboelhassan R., El Wakeel L.M. (2020). Evaluation of the effect of N-acetylcysteine on the prevention and amelioration of paclitaxel-induced peripheral neuropathy in breast cancer patients: A randomized controlled study. Breast Cancer Res. Treat..

[138] Bondad N., Boostani R., Barri A., Elyasi S., Allahyari A. (2020). Protective effect of N-acetylcysteine on oxaliplatin-induced neurotoxicity in patients with colorectal and gastric cancers: A randomized, double blind, placebo-controlled, clinical trial. J. Oncol. Pharm. Pract..

[139] Spector R., Johanson C.E. (2007). Vitamin transport and homeostasis in mammalian brain: Focus on Vitamins B and E. J. Neurochem..

[140] Hoehler D., Marquardt R.R. (1996). Influence of vitamins E and C on the toxic effects of ochratoxin A and T-2 toxin in chicks. Poult. Sci..

[141] Rizzo A.F., Atroshi F., Ahotupa M., Sankari S., Elovaara E. (1994). Protective effect of antioxidants against free radical-mediated lipid peroxidation induced by DON or T-2 toxin. Zentralblatt fur. Vet. Reihe A.

[142] Ning C., Xiao W., Liang Z., Wu Y., Fan H., Wang S., Kong X., Wang Y., Wu A., Li Y. (2024). Melatonin alleviates T-2 toxin-induced oxidative damage, inflammatory response, and apoptosis in piglet spleen and thymus. Int. Immunopharmacol..

[143] Atroshi F., Rizzo A., Biese I., Veijalainen P., Antila E., Westermarck T. (1997). T-2 toxin-induced DNA damage in mouse livers: The effect of pretreatment with coenzyme Q10 and alpha-tocopherol. Mol. Asp. Med..

[144] Tinkov A.A., Skalny A.V., Guo X., Korobeinikova T.V., Ning Y., Rocha J.B.T., Zhang F., Aschner M. (2025). Review of the Protective Effects of Selenium against T-2 Toxin-Induced Toxicity. Chem. Res. Toxicol..

[145] Moosavi M., Rezaei M., Kalantari H., Behfar A., Varnaseri G. (2016). l-carnitine protects rat hepatocytes from oxidative stress induced by T-2 toxin. Drug Chem. Toxicol..

[146] Argyriou A.A., Chroni E., Koutras A., Iconomou G., Papapetropoulos S., Polychronopoulos P., Kalofonos H.P. (2006). A randomized controlled trial evaluating the efficacy and safety of vitamin E supplementation for protection against cisplatin-induced peripheral neuropathy: Final results. Support. Care Cancer.

[147] Sarraf N., Badri T., Keshvari N., Ghassab-Sahebkar A., Qobadighadikolaei R., Abbasinazari M. (2020). Comparison of the efficacy and safety of melatonin and memantine in the alleviation of cognitive impairments induced by electroconvulsive therapy: A randomized clinical trial. J. Clin. Neurosci..

[148] Johnston D.L., Zupanec S., Nicksy D., Morgenstern D., Narendran A., Deyell R.J., Samson Y., Wu B., Baruchel S. (2019). Phase I dose-finding study for melatonin in pediatric oncology patients with relapsed solid tumors. Pediatr. Blood Cancer.

[149] Zhao Y., Valis M., Wang X., Nepovimova E., Wu Q., Kuca K. (2024). HIF-1α is a “brake” in JNK-mediated activation of amyloid protein precursor and hyperphosphorylation of tau induced by T-2 toxin in BV2 cells. Mycotoxin Res..

[150] Liu X., Wang Z., Wang X., Yan X., He Q., Liu S., Ye M., Li X., Yuan Z., Wu J. (2021). Involvement of endoplasmic reticulum stress-activated PERK-eIF2α-ATF4 signaling pathway in T-2 toxin-induced apoptosis of porcine renal epithelial cells. Toxicol. Appl. Pharmacol..

[151] Yi Y., Zhao F., Wang N., Liu H., Yu L., Wang A., Jin Y. (2018). Endoplasmic reticulum stress is involved in the T-2 toxin-induced apoptosis in goat endometrium epithelial cells. J. Appl. Toxicol..

[152] Cornelissen J., Kirtland S., Lim E., Goddard M., Bellm S., Sheridan K., Large S., Vuylsteke A. (2006). Biological efficacy of low against medium dose aspirin regimen after coronary surgery: Analysis of platelet function. Thromb. Haemost..

[153] Paganoni S., Macklin E.A., Hendrix S., Berry J.D., Elliott M.A., Maiser S., Karam C., Caress J.B., Owegi M.A., Quick A. (2020). Trial of Sodium Phenylbutyrate-Taurursodiol for Amyotrophic Lateral Sclerosis. N. Engl. J. Med..

[154] Paganoni S., Hendrix S., Dickson S.P., Knowlton N., Macklin E.A., Berry J.D., Elliott M.A., Maiser S., Karam C., Caress J.B. (2021). Long-term survival of participants in the CENTAUR trial of sodium phenylbutyrate-taurursodiol in amyotrophic lateral sclerosis. Muscle Nerve.

[155] Bahi A., Dreyer J.L. (2019). Dopamine transporter (DAT) knockdown in the nucleus accumbens improves anxiety- and depression-related behaviors in adult mice. Behav. Brain Res..

[156] Silva R.F.M., Pogačnik L. (2020). Polyphenols from Food and Natural Products: Neuroprotection and Safety. Antioxidants.

[157] Dai C., Xiao X., Li J., Ciccotosto G.D., Cappai R., Tang S., Schneider-Futschik E.K., Hoyer D., Velkov T., Shen J. (2019). Molecular Mechanisms of Neurotoxicity Induced by Polymyxins and Chemoprevention. ACS Chem. Neurosci..

[158] Del Fabbro L., Sari M.H.M., Ferreira L.M., Furian A.F. (2024). Natural compounds mitigate mycotoxins-induced neurotoxicity by modulating oxidative tonus: In vitro and in vivo insights—A review. Food Addit. Contam. Part A Chem. Anal. Control Expo. Risk Assess..

[159] Gugliandolo E., Peritore A.F., D’Amico R., Licata P., Crupi R. (2020). Evaluation of Neuroprotective Effects of Quercetin against Aflatoxin B1-Intoxicated Mice. Animals.

[160] Linardaki Z.I., Lamari F.N., Margarity M. (2017). Saffron (*Crocus sativus* L.) Tea Intake Prevents Learning/Memory Defects and Neurobiochemical Alterations Induced by Aflatoxin B(1) Exposure in Adult Mice. Neurochem. Res..

[161] Zhu L., Yi X., Ma C., Luo C., Kong L., Lin X., Gao X., Yuan Z., Wen L., Li R. (2020). Betulinic Acid Attenuates Oxidative Stress in the Thymus Induced by Acute Exposure to T-2 Toxin via Regulation of the MAPK/Nrf2 Signaling Pathway. Toxins.

[162] Wu J., Yang C., Liu J., Chen J., Huang C., Wang J., Liang Z., Wen L., Yi J.E., Yuan Z. (2019). Betulinic Acid Attenuates T-2-Toxin-Induced Testis Oxidative Damage Through Regulation of the JAK2/STAT3 Signaling Pathway in Mice. Biomolecules.

[163] Li X., Wang X., Liu S., Wang J., Liu X., Zhu Y., Zhang L., Li R. (2021). Betulinic acid attenuates T-2 toxin-induced cytotoxicity in porcine kidney cells by blocking oxidative stress and endoplasmic reticulum stress. Comp. Biochem. Physiol. Toxicol. Pharmacol..

[164] Kong L., Zhu L., Yi X., Huang Y., Zhao H., Chen Y., Yuan Z., Wen L., Wu J., Yi J. (2021). Betulinic Acid Alleviates Spleen Oxidative Damage Induced by Acute Intraperitoneal Exposure to T-2 Toxin by Activating Nrf2 and Inhibiting MAPK Signaling Pathways. Antioxidants.

[165] Huang L., Zhu L., Ou Z., Ma C., Kong L., Huang Y., Chen Y., Zhao H., Wen L., Wu J. (2021). Betulinic acid protects against renal damage by attenuation of oxidative stress and inflammation via Nrf2 signaling pathway in T-2 toxin-induced mice. Int. Immunopharmacol..

[166] Luo C., Huang C., Zhu L., Kong L., Yuan Z., Wen L., Li R., Wu J., Yi J. (2020). Betulinic Acid Ameliorates the T-2 Toxin-Triggered Intestinal Impairment in Mice by Inhibiting Inflammation and Mucosal Barrier Dysfunction through the NF-κB Signaling Pathway. Toxins.

[167] Huang C., Ou Z., Kong L., Huang Y., Yang W., He J., Yang M., Wu J., Xiang S., Zhou Y. (2024). Betulinic acid attenuates T-2 toxin-induced lung injury by activating Nrf2 signaling pathway and inhibiting MAPK/NF-κB signaling pathway. Toxicon.

[168] Moussa C., Hebron M., Huang X., Ahn J., Rissman R.A., Aisen P.S., Turner R.S. (2017). Resveratrol regulates neuro-inflammation and induces adaptive immunity in Alzheimer’s disease. J. Neuroinflammation.

[169] Liu X., Baxley S., Hebron M., Turner R.S., Moussa C. (2025). Resveratrol Attenuates CSF Markers of Neurodegeneration and Neuroinflammation in Individuals with Alzheimer’s Disease. Int. J. Mol. Sci..

[170] Singh T., Thapliyal S., Bhatia S., Singh V., Singh M., Singh H., Kumar A., Mishra A. (2022). Reconnoitering the transformative journey of minocycline from an antibiotic to an antiepileptic drug. Life Sci..

[171] Lu Y., Zhou M., Li Y., Li Y., Hua Y., Fan Y. (2021). Minocycline promotes functional recovery in ischemic stroke by modulating microglia polarization through STAT1/STAT6 pathways. Biochem. Pharmacol..

[172] Thomas M., Le W.D., Jankovic J. (2003). Minocycline and other tetracycline derivatives: A neuroprotective strategy in Parkinson’s disease and Huntington’s disease. Clin. Neuropharmacol..

[173] Wang Y., Nie D., Shao K., Zhang S., Wang Q., Han Z., Chen L. (2024). Mechanistic insights into the parental co-exposure of T-2 toxin and epoxiconazole on the F1 generation of zebrafish (Danio rerio). Chemosphere.

[174] Wattanasuntorn P., Poapolathep S., Phuektes P., Alassane-Kpembi I., Fink-Gremmels J., Oswald I.P., Poapolathep A. (2025). Apoptotic Effect of Combinations of T-2, HT-2, and Diacetoxyscirpenol on Human Jurkat T Cells. Toxins.

[175] Lin X., Shao W., Yu F., Xing K., Liu H., Zhang F., Goldring M.B., Lammi M.J., Guo X. (2019). Individual and combined toxicity of T-2 toxin and deoxynivalenol on human C-28/I2 and rat primary chondrocytes. J. Appl. Toxicol..

